# Between a rock and a soft place: Using optical ages to date ancient clam gardens on the Pacific Northwest

**DOI:** 10.1371/journal.pone.0171775

**Published:** 2017-02-09

**Authors:** Christina M. Neudorf, Nicole Smith, Dana Lepofsky, Ginevra Toniello, Olav B. Lian

**Affiliations:** 1 Department of Geography and the Environment, University of the Fraser Valley, Abbotsford, BC, Canada; 2 Department of Geography, Simon Fraser University, Burnaby, BC, Canada; 3 Hakai Institute, Heriot Bay, BC, Canada; 4 Department of Archaeology, Simon Fraser University, Burnaby, BC, Canada; Heidelberg University, GERMANY

## Abstract

Rock-walled archaeological features are notoriously hard to date, largely because of the absence of suitable organic material for radiocarbon dating. This study demonstrates the efficacy of dating clam garden wall construction using optical dating, and uses optical ages to determine how sedimentation rates in the intertidal zone are affected by clam garden construction. Clam gardens are rock-walled, intertidal terraces that were constructed and maintained by coastal First Nation peoples to increase bivalve habitat and productivity. These features are evidence of ancient shellfish mariculture on the Pacific Northwest and, based on radiocarbon dating, date to at least the late Holocene. Optical dating exploits the luminescence signals of quartz or feldspar minerals to determine the last time the minerals were exposed to sunlight (i.e., their burial age), and thus does not require the presence of organic material. Optical ages were obtained from three clam garden sites on northern Quadra Island, British Columbia, and their reliability was assessed by comparing them to radiocarbon ages derived from shells underneath the clam garden walls, as well as below the terrace sediments. Our optical and radiocarbon ages suggest that construction of these clam garden walls commenced between ~1000 and ~1700 years ago, and our optical ages suggest that construction of the walls was likely incremental and increased sedimentation rates in the intertidal zone by up to fourfold. Results of this study show that when site characteristics are not amenable to radiocarbon dating, optical dating may be the only viable geochronometer. Furthermore, dating rock-walled marine management features and their geomorphic impact can lead to significant advances in our understanding of the intimate relationships that Indigenous peoples worldwide developed with their seascapes.

## Introduction

Rock-walled archaeological features, such as fish traps and agricultural terraces, have the potential to provide rich insights into past relationships between people and their natural worlds, and how those relationships developed through time. However, while such features are common in the archaeological record [1–5] their interpretive significance is often limited by how difficult it is to date when they were constructed [6, 7]. Ages of these features have been determined using optical or radiocarbon ages of material in sediment fills [8–10], lichenometry of rock surfaces [11], association with the age of cultural material or settlements in the vicinity of the features [12, 2, 13], and the masonry style of wall construction [14, 15]. In the case of dated materials in terrace fills, reliable ages can be limited by cultivation that mixes deposits, post-depositional processes that generate sheet or rill wash, bioturbation, and dating organic or cultural material that is inherited from pre-existing deposits used to construct the terrace [14]. Given these potential issues, the best approach for dating rock-walled features is to employ more than one technique to provide bracketing, if not firm, ages for wall construction and use [16].

Along the Pacific Northwest coast, rock-walled beach terraces locally known as “clam gardens” are evidence of an ancient system of cultivation of clams and other marine resources. Such features are known from Alaska, British Columbia (BC), and Washington State, and are a major focus of ecological, archaeological, and anthropological research [17–20]. Coastal First Nations constructed rock walls in the lowest intertidal zone to trap loose sediments, thus creating or expanding clam habitat and increasing bivalve production. An outstanding question in clam garden research is whether the terrace walls were built to a certain tidal height on initial construction, or whether the walls were built up incrementally over time by rolling rocks while harvesting. We know from the Indigenous terms for these features, as well as from local First Nations knowledge holders, that rolling rocks downslope was part of the regular traditional management of productive clam beaches [19, 20]. Determining when these features were constructed, and understanding the timing and nature of the terrace infilling process will help us understand the temporal relationships between settlement patterns of ancient peoples and terrace construction, and therefore, the history of Indigenous subsistence strategies and land use along the coast.

While clam gardens and other rock-walled features (e.g., fish traps and stone-faced root gardens [20]) are a major component of the region’s archaeological record, archaeologists’ ability to date these subsistence features is often limited to the chance retrieval of organic material associated with the placement of the stones at the time of construction. In the case of clam gardens, valid radiocarbon ages have been obtained from ancient barnacle scars preserved on the rock wall and from the shells of clams trapped by wall construction. Unfortunately, however, both methods are limited to fairly specific depositional contexts. Furthermore, marine shells can survive on the surface of a beach for millennia after the organism died [19]. Thus, a shell trapped by the wall or terrace infill sediments may not be a valid indicator of the time of wall construction, but rather, pre-date it by several thousand years.

This is the first study to use optical dating to determine when clam garden walls were initially constructed and to assess the timing of the subsequent infilling of the terrace. As such, we present an alternative to radiocarbon dating and expand the instances in which clam gardens can be dated. Our assessment of infilling rates provides much needed insights into the intentions and on-going relationships of people to these managed clam beaches. The robustness of our optical dating procedures is assessed by comparing our optical age estimates with radiocarbon ages derived from shells underneath the clam garden walls, as well as below the terrace infill. We show that infrared stimulated luminescence (IRSL) signals from terrace sediments can detect changes in sedimentation rates that reflect not only the time, but also the rate of clam garden wall construction and build-up. Our methods have the potential to be applied to other archaeological rock-walled features to enhance our understanding of the myriad of ways in which Indigenous peoples interacted with their land and seascapes.

## Study sites

**Fig 1:**
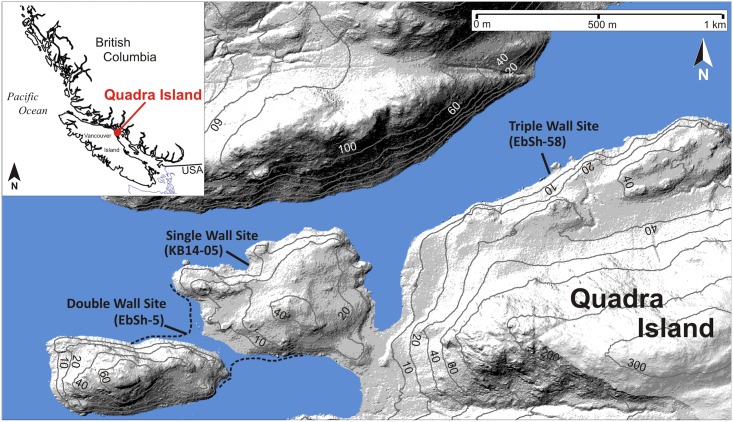
Locations of clam garden study sites superimposed on a hillshaded and contoured LiDAR digital elevation model (average point density of 18.2 pts/m^2^). The LiDAR data were obtained and processed by Rob Vogt of the University of Northern British Columbia Lidar Research Group and Derek Heathfield of the Hakai Institute Coastal Sand Ecosystems program.

Northern Quadra Island, southwest BC (Fig 1) has among the highest density of clam gardens on the Pacific Northwest coast. Clam gardens on Quadra Island are being studied by a team of archaeologists and ecologists who are interested in the social and ecological impacts of building clam gardens in the past and in the present (www.clamgarden.com). The gardens occur in a variety of forms and geomorphic settings, but are most commonly found away from deltas, along the sides of semi-protected inlets with strong tidal currents. Some clam garden walls were created by pushing rocks downslope on a narrow strip of land along a bedrock shoreline, some by placing rocks on natural intertidal rocky platforms or ridges, while others were built by stacking rocks on sloping sandy and gravelly substrates. All forms have walls that were built at the position of the lowest low tide relative to the sea level of the time to maximize the formation of ideal clam habitat. Some rock walls have well-built foundations, while others are less structured. In all cases, people seem to have subsequently augmented and maintained these initial walls, probably as they were clearing the beach of rocks while digging for clams [20].

**Fig 2:**
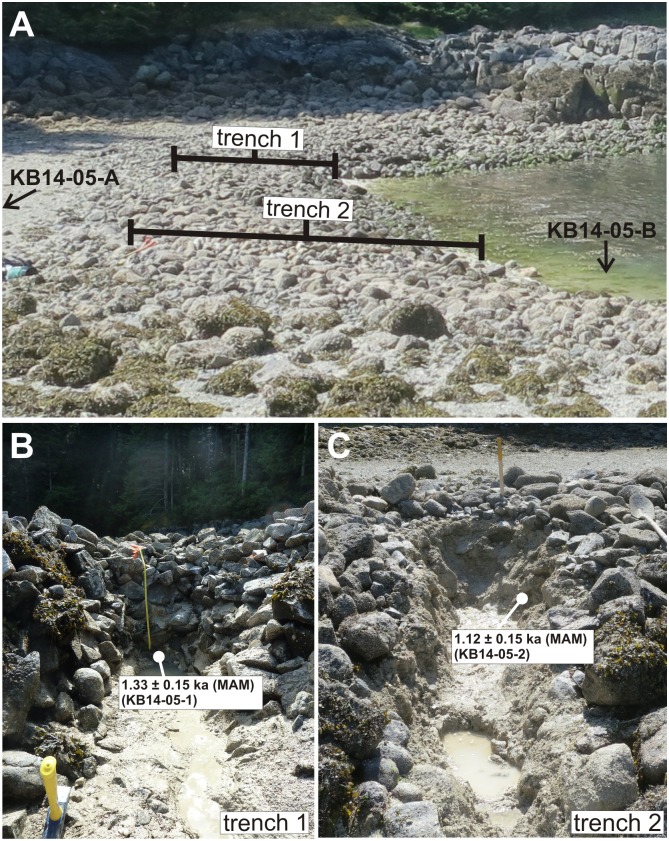
A) Location of trenches 1 and 2 at Single Wall Site, KB14-05. Vertical core KB14-05-B was extracted from non-walled beach sediments seaward of trench 2 (arrow). Vertical core KB14-05-A was extracted from terrace sediments ~2 m inland from the edge of the photo (arrow). B) and C) show the locations of optical samples KB14-05-1 and KB14-05-2. Sample KB14-05-1 was collected from sediments immediately underlying the lowest rocks of the clam garden wall in trench 1. Sample KB14-05-2 was collected from sediments between rocks within the clam garden wall, 50 cm below the terrace surface in trench 2.

**Fig 3:**
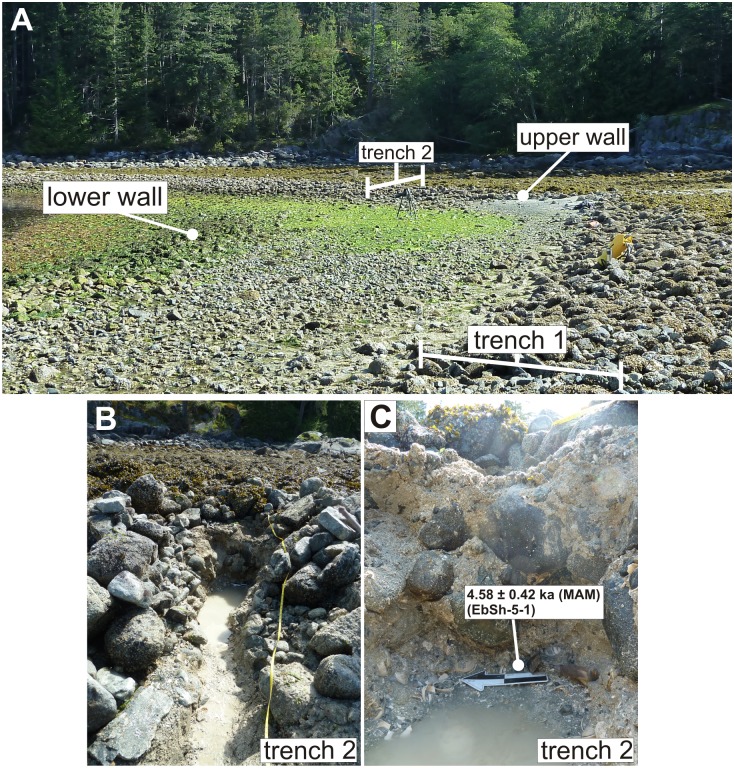
A) Location of trenches 1 and 2 at Double Wall Site, EbSh-5. The best maximum age estimate for wall construction was obtained from a radiocarbon age of 980–1362 cal years BP from a *Saxidomus gigantea* shell collected from below the wall in trench 1. Optical sample EbSh-5-1 was taken from sediments immediately below the upper wall in trench 2. The Minimum Age Model (MAM) age of this sample is consistent with a radiocarbon age of 3619–3969 cal years BP obtained from a *S*. *gigantea* shell immediately next to the optical dating sample. The correspondence between the MAM-based age and the radiocarbon age determinations gives us confidence in our optical dating methods.

**Fig 4:**
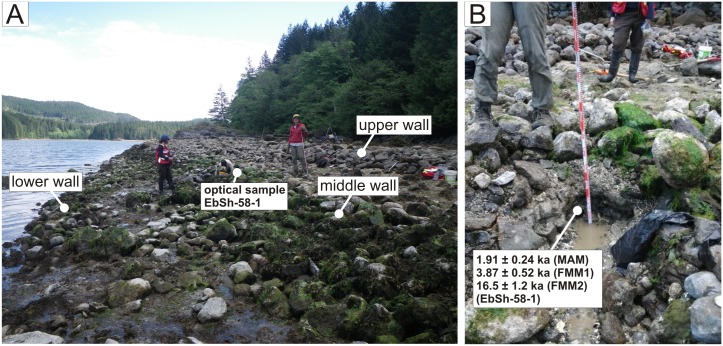
A) Triple Wall Site, EbSh-58. Optical sample EbSh-58-1 was taken from sediments immediately below the middle wall. The ages of the youngest aliquots measured from this sample (i.e., the Minimum Age Model age of 1.43–2.40 ka) is consistent with a radiocarbon age of 1279–1673 cal years BP from a *Nucella sp*. shell collected from the surface of the pre-garden beach underlying the highest terrace, suggesting that the pre-garden beach surface underlying both the middle and upper walls is ~1200–2400 years old. These ages provide the best maximum age estimates of wall construction on the middle and highest terraces. The youngest Finite Mixture Model (FMM) component (2.83–4.91 ka) is consistent with a pre-wall construction radiocarbon age of 3336–3799 cal years BP from a *S*. *gigantea* shell in growth position found trapped below the middle wall. The older FMM component (16.5 ± 1.2 ka) dates back to early postglacial time, and likely records the deposition of outwash sand by glaciers proximal to site EbSh-58.

In this study, three clam gardens on northern Quadra Island were sampled for radiocarbon and optical dating (sites KB14-05, EbSh-5 and EbSh-58) (Fig 1). Each of these gardens was formed by constructing rock walls on sloping sandy and gravelly beaches. Site KB14-05 contains a single wall that rises to an elevation of 2.27 m above present day lowest low water large tide (LLWLT) (Fig 2). In this region, LLWLT is a CGVD28 (or Canadian Geodetic Vertical Datum of 1928) height of -2.41 m and 0.06 m below chart datum, or the level below which the water surface seldom falls [21]. Site EbSh-5 contains two walls, the lowest rising to 1.35 m and the highest rising to 2.52 m above LLWLT (Fig 3). The foundation of the upper wall at site EbSh-5 was built with interlocking angular boulders, upon which more rounded stones were placed, presumably during construction and on-going maintenance [19]. Site EbSh-58 consists of three walls that rise to elevations of 0.63 m, 1.48 m, and 2.09 m above LLWLT (Fig 4). The clam garden walls at all sites are composed of pebbles, cobbles, and boulders in a sandy, shell-rich matrix. The sediment infill landward of the walls is pebbly medium-coarse sand with shell hash concentrations of ~10–50%. At all sites, we collected radiocarbon dates from shells below the walls or below the terrace infill to provide independent assessments of the ages of wall construction and maintenance.

## Methods

### Sampling strategy

We had two objectives for the optical dating of the Quadra Island clam gardens: 1) to estimate the time of initial clam garden wall construction and on-going use, and 2) to estimate the time and rate of infilling of sediments behind the clam garden walls; these provide an indirect indicator of how quickly the wall was constructed and how human modification of the landscape influenced sedimentation rates in the intertidal zone. Sampling was conducted with permission from the Province of British Columbia, Heritage Conservation Act Permit #2014–0095.

**Fig 5:**
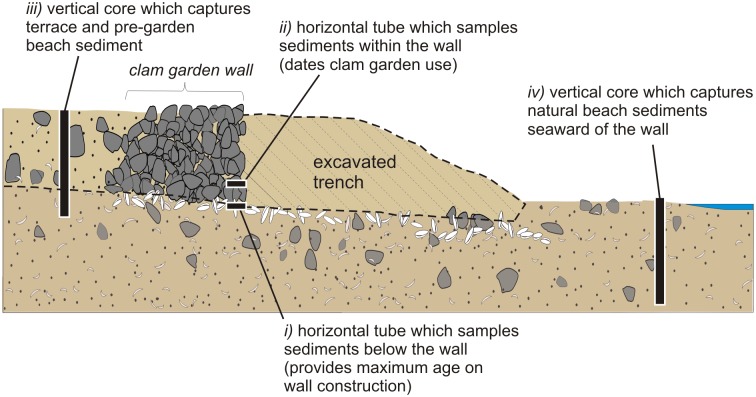
A conceptual diagram illustrating the sampling strategy for optical dating clam gardens. All methods (*i–iv*) were applied at site KB14-05 (Single Wall Site) while one method (*i*) was applied at sites EbSh-5 (Double Wall Site) and EbSh-58 (Triple Wall Site). Not drawn to scale.

To achieve these objectives, we used three sampling methods: *i*) hammering small (20 cm long, 4 cm diameter) steel tubes horizontally into sediments beneath the clam garden walls and extracting them in an attempt to obtain a maximum age of wall construction (Objective 1; samples KB14-05-1 and 2, EbSh-5-1, EbSh-58-1). *ii*) Hammering a similar sized tube horizontally into sediments between the rocks within one clam garden wall and extracting it to provide a midway age of garden use (Objective 1) and to check the method by comparing this age with those below it (KB14-05-2). *iii*) Hammering a PVC or ABS pipe (~1 m long, 4 cm in diameter) vertically into the top of the terrace behind (landward) of one wall and extracting it to provide age estimates of the pre-garden beach surface (Objective 1), and to estimate the time and rate of sediment infilling behind the wall (Objective 2; KB14-05-A), and *iv*) hammering a similar sized pipe vertically into non-walled beach sand in front (seaward) of a wall and extracting it to compare the sedimentation rate of non-walled beach sediments to that of the clam garden terrace sediments (Objective 2; KB14-05-B) (Fig 5). Where possible, all optical sampling was paired with the collection of radiocarbon samples. All methods (*i–iv*) were applied at site KB14-05 (Single Wall Site) while only one method (*i*) was applied at sites EbSh-5 (Double Wall Site) and EbSh-58 (Triple Wall Site). After extraction, both ends of the steel tubes and pipes were sealed promptly with opaque black plastic and duct tape and transported to the Luminescence Dating Laboratory at the University of the Fraser Valley for processing.

### Laboratory preparation

#### Optical dating

Optical dating is a method that determines the last time mineral grains (in this case, sand sized potassium-rich feldspar (Ksp) grains) were exposed to sunlight, where the calibrated natural luminescence signal measured from individual grains or multi-grain aliquots is used to determine an ‘equivalent dose’ (D_e_). This is a laboratory estimate of the total radiation absorbed by those grains since burial [22]. The D_e_ value of a sample (usually derived from statistical analysis of a distribution of D_e_ values obtained from individual aliquots, each consisting of a single grain or multiple grains) is divided by the environmental dose rate at the sampling site to obtain an optical age [23]. The thermally-stable optical signal in feldspar is well known to fade over time (a process known as anomalous fading) [24]. Thus, the rate of fading must also be measured in the laboratory so that corrections can be applied to calculated ages.

In the laboratory, sediments were extracted from the small steel tubes and the PVC/ABS pipes under dim orange light. The outermost (light-exposed) portions of each tube/pipe were retained for water content measurements and for the determination of radionuclide (K, Th, U, Rb) concentrations (see Supporting Information for details). Ksp grains were concentrated for optical dating using standard density separation procedures. This was done because laboratory experiments showed that, unlike Ksp, luminescence signals from quartz are too dim for dating. See Supporting Information for further details on optical sample preparation.

#### Interpreting D_e_ distributions

Evidence for insufficient sunlight exposure of grains prior to burial, or mixing of sediment grains between layers of different age in a sample, can be inferred from the shape and amount of scatter in its D_e_ distribution (referred to as overdispersion [25]). ‘Overdispersion’ (OD) is calculated using the Central Age Model (CAM) and is the spread in D_e_ values relative to the weighted mean (also calculated using CAM) that remains after all measurement uncertainties have been taken into account [25, 26]. OD can be the result of incomplete bleaching and sediment mixing, or, in complex depositional environments, differences in β doses received by individual grains due to their proximity to pore water, cemented grain clusters, rocks or organic material with different dosimetric characteristics [27–30].

In this study, we expected that samples extracted from non-walled sediments deposited by shallow, low-energy waters in the intertidal zone on Quadra Island to have low to moderate OD values (~15–40%) that reflect partial bleaching of grains deposited subaqueously and some mixing by burrowing fauna. In samples collected from clam terrace sediments, with their increased bivalve production [18], we expected to see moderate to high OD values (>40%) that reflect more bioturbation from burrowing clams and mixing by humans during clam harvesting.

#### Vertical cores

The ~1 m long PVC and ABS pipes were cut open along their length with a Dremel tool and the sediments inside were photographed using filtered light. The sediments were sampled every 2 cm and treated with HCl acid (10%) and H_2_O_2_ (10%) to remove carbonates and dissolve organic material, respectively. The IRSL signal intensity from the remaining minerals, which was presumably mainly from the feldspar fraction, were measured in all samples and used as a proxy for relative changes in sedimentation rate. The sediments were compacted during sampling and comparisons between the depth of the sample hole, and the thickness of the retrieved sediments in the core showed that 2 cm of compacted sediment collected from the clam garden terrace equals ~4 cm of sediment before compaction, and 2 cm of compacted sediment collected from beach sediments seaward of the wall equals ~7.5 cm of sediment before compaction, assuming equal compression along the length of the cores.

The IRSL signal intensities measured from the cores were interpreted in the following ways: abrupt increases in down-core signal intensity were interpreted to represent an unconformity in the sedimentary record that reflects an erosional boundary or hiatus in sedimentation. Gradual increases in down-core signal intensity were interpreted as an indication of gradual sedimentation (i.e., a constant sedimentation rate). And finally, no, or minimal changes in down-core signal intensity were interpreted as an indication of rapid deposition and/or bioturbation and other processes of sediment mixing throughout the thickness of the sedimentary unit. After examining down-core changes in signal intensities, the cores were further subsampled at key positions and processed to concentrate Ksp for optical dating. These optical samples were used to quantify sedimentation rates and estimate the time of any abrupt changes in sedimentation rate. We expected to see high OD values, and possibly bimodal D_e_ distributions, in core subsamples extracted from areas exhibiting abrupt changes in down-core signal intensity (i.e., the contact between two sedimentary units), as it is likely that these samples would contain grains from sedimentary units of different ages.

#### Testing the reliability of our measurement procedures, and corrections for anomalous fading

The De values for Ksp-concentrated multi-grain aliquots were determined using a single aliquot regenerative-dose (SAR) protocol [31] (Table B in S1 Supporting Information). This protocol has been applied successfully to other samples on the coast of BC [32] and its suitability to date Ksp grains from Quadra Island was confirmed using a dose recovery test. Measurements of fading rates have been made and corrections for fading have been applied to all dated samples. See Supporting Information for further information on D_e_ determination, dose recovery testing, and fading measurements and corrections.

## Results

The radiocarbon ages (Table 1) in combination with the optical age estimates (Tables 2 and 3) from the three clam garden sites provide a powerful means to estimate when the clam garden walls were constructed and used. Below we identify the ages that provided the best estimates for the time of wall construction (Objective 1) and show how optical ages helped us calculate human induced changes in sedimentation rate (Objective 2). Optical ages helped us constrain the time of initial construction of the clam garden wall at the Single and Triple Wall sites (KB14-05 and EbSh-58), as well as the terrace sedimentation rate and the time of terrace infilling at the Single Wall Site (KB14-05). Despite attempts to sample sand grains immediately under the walls, some optical ages serve as poor constraints for the time of wall construction as they date older pre-garden beach sediments that were deposited a few thousand years before the event.

**Table 1:** Radiocarbon ages from excavations of three clam gardens on Quadra Island, BC (2013–2015).

Site	Radiocarbon dating sample context	UCIAMS	Material	^14^C age (yrs BP)	Calibrated age^1^ (cal yrs BP)	Interpretation
KB14-05 (single wall)	Clam shell in growth position in sediments immediately under the wall, 82 cm below the terrace surface (trench 1).	141821	*Saxidomus gigantea* shell	4165 ± 20	3567–4061	Provides an age for the pre-garden beach and a maximum age for wall construction.
EbSh-5 (double wall)	Clam shell in pre-garden beach sediments immediately below the wall, 50–55 cm below the highest terrace surface (trench 1).	175684	*Saxidomus gigantea* shell	1960 ± 15	980–1362	Provides an age for the pre-garden beach and the best maximum age for wall construction.
	Clam shell from pre-garden beach sediments immediately underlying the wall and adjacent to optical dating sample EbSh-5-1, 90 cm below the surface of the highest terrace (trench 2).	141816	*Saxidomus gigantea* shell	4260 ± 20	3677–4198	Provides an age for the pre-garden beach and a maximum age for wall construction.
EbSh-58 (triple wall)	Whelk on pre-garden beach surface, 40 cm below surface of highest terrace.	141817	*Nucella sp*. shell	2225 ± 20	1279–1673	Provides an age for the pre-garden beach surface and the best maximum age for wall construction.
	Clam shell on pre-garden beach surface below the middle wall, 40 cm below the middle wall terrace surface.	141815	*Saxidomus gigantea* shell	3955 ± 25	3336–3799	Provides an age for the pre-garden beach surface and a maximum age for wall construction.

^1^ All ages were calibrated at 2σ using Calib 7.0 with the Marine 13 calibration curve using a marine reservoir correction of 320 + 90 years for post-10,000 ^14^C year BP samples [33].

**Table 2:** Optical ages from three clam gardens on Quadra Island, BC (2014–2015). See Table 3 for more information.

Site	Context	Sample name	Age^1^ (ka)	Interpretation
Small tube samples				
KB14-05 (single wall)	Pre-garden beach sediments immediately underlying the wall (trench 1).	KB14-05-1	1.33 ± 0.15	The best maximum age for wall construction.
	Sediments within the wall, 50 cm below the terrace surface (trench 2).	KB14-05-2	1.12 ± 0.15	Post-wall construction age (dates wall use).
EbSh-5 (double wall)	Sediments immediately underlying the highest wall, 90 cm below the terrace surface (trench 2).	EbSh-5-1	4.58 ± 0.42	Dates a period of pre-garden beach deposition before wall construction.
EbSh-58 (triple wall)	Sediments immediately underlying the middle wall, 30 cm below the surface.	EbSh-58-1	1.91 ± 0.24 (MAM)	Dates earliest possible time of wall construction (MAM), late Holocene pre-garden beach deposition (FMM1) before wall construction and early post-glacial sand deposition (FMM2).
			3.87 ± 0.52 (FMM1)	
			16.5 ± 1.2 (FMM2)	
Vertical core samples				
KB14-05 (single wall)	Terrace and underlying pre-garden beach sediments 7.2 m inland from the crest of the wall.	KB14-05-A1	0.51 ± 0.14 (FMM1)	Represents pre-garden beach sand deposition (FMM1) followed by sedimentation behind the clam garden wall (FMM2). Sediments behind the wall were likely re-exposed to sunlight during human harvesting and bioturbation.
			1.68 ± 0.16 (FMM2)	
		KB14-05-A2	4.09 ± 0.37	Dates pre-garden beach deposition before wall construction.
		KB14-05-A3	2.88 ± 0.32	Dates pre-garden beach deposition before wall construction.
		KB14-05-A4	5.46 ± 0.55	Dates pre-garden beach deposition before wall construction.
	Non-walled beach sediments 3.30 m seaward of the wall.	KB14-05-B1	0.18 ± 0.02	Represents deposition of non-walled beach sediments.
		KB14-05-B2	9.30 ± 0.81	Represents deposition of non-walled beach sediments.

^1^ MAM refers to Minimum Age Model, and FMM1 and FMM2 refer to Finite Mixture Model component ages 1 and 2, respectively. See text and Table 3 for explanation. Errors are ± 1 σ.

**Table 3:** Sample D_e_ values, OD values, fading rates and optical ages^1^. Reported fading rates are weighted mean values from 12 multi-grain aliquots measured from each sample.

Sample (UFV Laboratory ID)	Total dose rate ^2^ (Gy/ka)	Number of aliquots measured	Number of aliquots accepted	D_e_ (CAM) (Gy)	OD (%)	Uncorrected age (ka)	*g*-value (%/decade)	CAM Fading-corrected age (ka)	MAM Fading-corrected age-outliers removed (ka)	FMM Fading-corrected ages (ka)
KB14-05-1(KB1405c)	1.46 ± 0.10	48	28	1.50 ± 0.09	31 ± 4	1.02 ± 0.14	6.53 ± 0.19	1.84 ± 0.18	1.33 ± 0.15	
KB14-05-1(KB1405c)^3^	1.43 ± 0.08	48	28	1.50 ± 0.09	31 ± 4	0.92 ± 0.13	6.53 ± 0.19	1.64 ± 0.17	1.34 ± 0.16	
KB14-05-2(Quad10)	1.78 ± 0.16	24	22	1.84 ± 0.13	34 ± 5	1.03 ± 0.12	4.16 ± 0.16	1.47 ± 0.17	1.12 ± 0.15	
KB14-05-A1(Quad5u)	1.90 ± 0.13	48	34	1.14 ± 0.13	66 ± 8	0.60 ± 0.08	6.82 ± 0.16	1.09 ± 0.15	0.54 ± 0.05	0.51 ± 0.14 1.68 ± 0.16
KB14-05-A2(Quad5x)	2.07 ± 0.15	24	23	7.04 ± 0.28	19 ± 3	3.40 ± 0.27	4.17 ± 0.15	4.96 ± 0.41	4.09 ± 0.37	
KB14-05-A3^3^^,^^4^^,^^5^(Quad5z)	1.78 ± 0.12	24	23	3.66 ± 0.24	31 ± 5	2.06 ± 0.19	not measured	3.94 ± 0.39	2.88 ± 0.32	
KB14-05-A4^3^^,^^4^(Quad5z8)	1.78 ± 0.12	24	21	5.94 ± 0.29	21 ± 4	3.35 ± 0.28	not measured	6.55 ± 0.57	5.46 ± 0.55	
KB14-05-B1(Quad9-16)	1.90 ± 0.12	71	22	0.39 ± 0.06	67 ± 11	0.21 ± 0.03	8.04 ± 0.17	0.40 ± 0.06	0.18 ± 0.02	
KB14-05-B2^4^(Quad9-03)	1.82 ± 0.13	24	21	7.27 ± 0.27	17 ± 3	4.01 ± 0.32	not measured	9.30 ± 0.81	N/A	
EbSh-5-1(KB07)	1.88 ± 0.13	24	21	5.36 ± 0.27	22 ± 4	2.86 ± 0.25	6.39 ± 0.17	5.28 ± 0.48	4.58 ± 0.42	
EbSh-58-1(TWB01)	1.91 ± 0.12	48	45	8.07 ± 1.29	106 ± 11	4.22 ± 0.73	7.07 ± 0.13	8.62 ± 1.50	1.91 ± 0.24	3.87 ± 0.52 16.5 ± 1.2

^1^ CAM, MAM and FMM are the Central Age Model, the Minimum Age Model, and the Finite Mixture Model, respectively [34, 35]. Because the MAM is sensitive to outliers in small datasets, MAM ages were calculated excluding the lowest outliers (see radial plots in Fig B in S1 Supporting Information).

^2^ Rb, U, Th and U concentrations were determined using neutron activation analysis (NAA) at Maxxam Analytics.

^3^ Rb, U, and Th concentrations were determined using neutron activation analysis (NAA), and the U was analyzed using delayed neutron counting at the Australian Nuclear Science and Technology Organisation (ANSTO), as this method can detect lower concentrations of U. Final age estimates using both methods are consistent with each other within error.

^4^ Due to limited sample sizes, fading rates of samples KB1405-A3, KB1405-A4 and KB1405-B2 could not be measured. The ages of samples KB1405-A3 and KB1405-A4 were corrected using the fading rate of KB1405-A1, and the age of sample KB1405-B2 was corrected using the fading rate of KB1405-B1.

^5^ An age inversion appears in core KB14-05-A (samples KB14-05-A2 and KB14-05-A3, Fig 7) and this may be due to: 1) the intrusion of young grains into sample KB14-05-A3 (more likely), or 2) to our inability to correct sample KB14-05-A3 for its own fading rate. Due to inadequate quantities of sample, fading measurements could not be conducted on KB14-05-A3, so its age was corrected using the fading rate of KB14-05-A1 (6.82 ± 0.16%/decade). If the fading rate of KB14-05-A3 was higher (e.g., 8–9%/decade), the age of this sample would be consistent with that of the overlying sample within 2 sigma. Because all but one of the fading rates determined from samples at this site are between 4.1 and 7.1%/decade, a higher fading rate for sample KB14-05-A3 is perhaps unlikely. The OD value of KB14-05-A3 is ~10% higher than that of both overlying and underlying samples in this core, and may reflect a higher proportion of young contaminating grains transported to this depth by burrowing fauna. Thus, the age inversion is best explained by bioturbation.

### Interpreting the D_e_ distributions

The OD values of all optical samples ranged between ~17 and ~106% with high (>60%) values probably reflecting a combination of incomplete bleaching of the signal in subaqueous environments and mixing of recently bleached grains with older sediments (see Discussion below). The OD values of most samples range between ~17 and ~40% and are typical of beach sediments from other parts of BC’s coast that have been dated using a similar method [32]. After observing the D_e_ distributions and OD values of all samples, D_e_ values for each sample were calculated using the CAM, the Minimum Age Model (MAM), and in two cases, the Finite Mixture Model (FMM) [34, 35].

The CAM was used to provide a weighted mean estimate of the D_e_ and assumes that all grains have been adequately bleached by sunlight prior to burial. The MAM was used to estimate the D_e_ of well-bleached grains in samples that were not bleached homogeneously before burial. Because our samples are from water lain deposits, we used the MAM to estimate the D_e_ values of all samples except sample KB14-05-B2, which has an OD value of only 17%, suggesting complete bleaching before burial. Because our measurements have been made on multi-grain aliquots, rather than on individual grains [30], our MAM-derived D_e_ values may still be larger than the D_e_ associated with the most recently bleached grains in our aliquots, as it is likely that some grains contributing to the signal measured from each of the aliquots will not have been completely bleached before burial. The FMM is designed for samples that consist of a mixture of grains from sedimentary units of different age [35]. This model was applied to two of our samples that have high OD values. These were collected from locations likely influenced by post-depositional mixing of sediments (KB14-05-A1 and EbSh-58-1).

### Objective 1: Estimating the time of initial clam garden wall construction and on-going use

Both optical and radiocarbon ages suggest that construction of clam garden walls at all sites commenced between ~1000 and ~1700 years ago. One optical age at the Single Wall Site indicates this wall was in use ~1100 years ago. In some cases, the age of the pre-garden beach sands is several thousand years older than the clam garden walls. In one instance at the Double Wall Site, and in two instances at the Triple Wall Site, optical age estimates agree with radiocarbon ages from shells from the same sediments, giving us confidence in our optical dating methodology.

#### EbSh-5 (Double Wall Site)

Our age estimates for the time of construction of the upper wall at the Double Wall Site highlight the difficulties of sampling pre-wall beach surfaces in the field. The youngest radiocarbon age estimate from site EbSh-5 suggests that the uppermost of the two clam garden walls was constructed sometime between ~1000 and ~1400 years ago, based on a *S*. *gigantea* shell retrieved from immediately below the wall in trench 1 (Table 1, sample 175684). Another *S*. *gigantea* shell from sand immediately underlying the wall in trench 2, ~25 m away, dates to ~3700–4200 years ago (Table 1, sample 141816). An optical sample obtained from sediments immediately adjacent to the older shell (sample EbSh-5-1, Table 2, Fig 3) gives a MAM age of 4.58 ± 0.42 ka (3.74–5.43 ka, at 2 sigma). The consistency between this optical age and the radiocarbon age from the same sediments gives us confidence in our optical dating methodology, but age estimates from both trenches raise a question about why we would retrieve two different dates immediately below the wall. Three possible explanations are: 1) the beach surface has been stable over time and the wall was built in stages (i.e., at ~3700–4200 and ~1000–1400 years ago), 2) the pre-garden beach surface experienced differential erosion across the width of the beach thereby removing the more recent 1000–3700 year old sediments and shells above sample 141816 (trench 2) prior to wall construction, and/or 3) relative sea level stability or slow regression during the late Holocene resulted in a relatively stable beach surface (i.e., sediment accumulation was negligible) and the accumulation of shells from the last ~4000 years on its surface prior to wall construction. Given a late Holocene age for wall construction is suggested from the radiocarbon and optical ages from other walls (see below), and that several other beaches have similarly aged bivalves likely associated with stable beach surfaces, we believe the third explanation is the most likely. Thus, based on the youngest radiocarbon age, this wall was built sometime after ~1000–1400 years.

Multi-grain aliquots of optical sample EbSh-5-1 do not appear to contain sediment grains from the pre-garden beach surface that were exposed to the sun just before the wall was built ~1000–1400 years ago, but rather sediment that was buried up to ~3700–5400 years before wall construction just below this surface (though single-grain dating is necessary to confirm this, see Discussion below). The preservation of such old sand grains so close to the base of the wall, and the observed mixture of young and old shells immediately below the wall from different locations, highlights the importance of collecting radiocarbon and horizontal optical dating samples from multiple contexts when trying to establish a clam garden age and, when possible, to partner the data with a vertical sediment core as described below.

#### EbSh-58 (Triple Wall Site)

Optical and radiocarbon ages at the Triple Wall Site suggest that the middle and uppermost clam garden walls were constructed sometime between ~1300 and ~2400 years ago on beach sands with sedimentary units dating back to ~3900 years and ~16,500 years. One MAM-based optical age estimate of 1.91 ± 0.24 ka (1.43–2.40 ka at 2 sigma) from sands immediately underlying the middle wall (sample EbSh58-1, Table 2) (Fig 4) best constrains the time of construction of the middle wall because it is the youngest below-wall optical age at this site and it is consistent with a radiocarbon age of 1279–1673 cal years BP from a *Nucella sp*. shell collected from the surface of pre-garden sediments below the highest terrace (Table 1).

**Fig 6:**
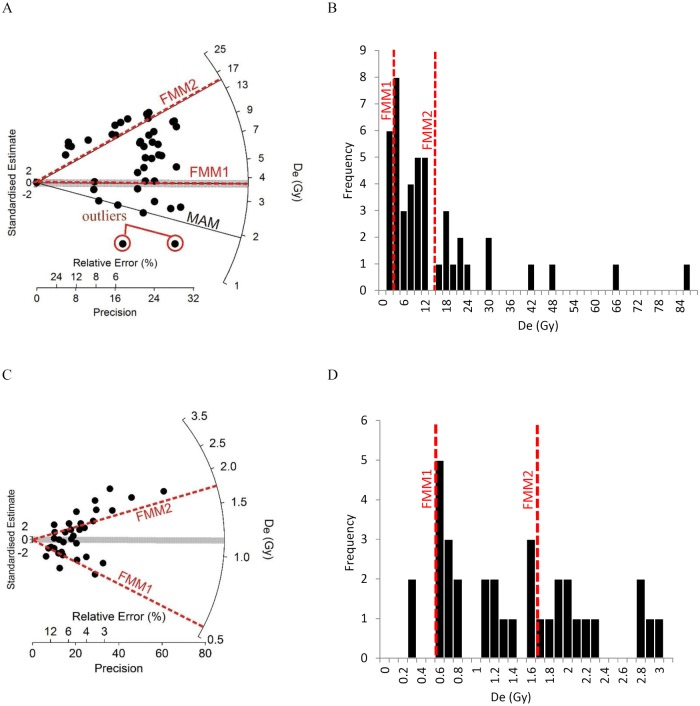
Multi-grain aliquot D_e_ distribution of samples EbSh-58-1 and KB14-05-A1. These samples are thought to contain mixtures of grains from sedimentary layers of different ages. The radial plot and histogram of EbSh-58-1 are plotted in (A) and (B), respectively. The radial plot and histogram of KB14-05-A1 are plotted in (C) and (D), respectively. Finite Mixture Model components 1 and 2 are shown as red dashed lines. The Minimum Age Model D_e_ value of sample EbSh-58-1 is shown as a solid black line, and was calculated after excluding two anomalously low D_e_ values (outliers).

As with sample EbSh-5-1 at the Double Wall Site, the high OD value of sample EbSh-58-1 suggested a mixed age D_e_ distribution and the presence of grains that were deposited long before wall construction. Thus, this sample was further analyzed using the FMM of Roberts et al. [35]. This model is used to identify objectively discrete D_e_ components in a sediment mixture. Following the approach of Jacobs et al. [36], the optimum number of D_e_ components and associated OD values are identified using two statistical parameters, the maximum log likelihood and Bayes information criterion. An assumption the FMM makes is that the optimum identified OD value is equal for all components. After considering the possible effects of aliquot-to-aliquot variations in fading rates on FMM calculations, and problems that can occur when applying the FMM to multi-grain aliquot D_e_ distributions (see Supporting Information), two components were identified. These give fading-corrected ages of 3.87 ± 0.52 ka (2.83–4.91 ka, at 2 sigma) and 16.5 ± 1.2 ka (14.1–18.9 ka, at 2 sigma), and high OD values (80%) suggestive of incomplete bleaching and bioturbation (Fig 6A and 6B, Tables 2 and 3).

The youngest FMM component (2.83–4.91 ka, at 2 sigma) is consistent with the radiocarbon age (3336–3799 cal years BP) of a *S*. *gigantea* shell found in growth position at the interface between the pre-garden beach surface and the sediment infill behind the middle wall (Table 1). This older clam shell and correspondingly old sand grains sampled immediately under the wall suggest that the pre-garden beach experienced limited aggradation for several thousand years prior to the construction of clam gardens during a period of relative sea level stability or slow regression. The oldest FMM component dates back to early postglacial time, and likely records outwash sand deposition by glaciers, the margins of which could have been proximal to site EbSh-58 ~16,500 years ago [37]. Such old sand grains so close to the base of the middle clam garden wall (and the lack of thick deposits of Holocene aged sediment) is evidence of either very low sedimentation rates or one or more periods of erosion at this location during the Holocene.

#### KB14-05 (Single Wall Site)

Optical ages from site KB14-05 suggest that the clam garden wall was built sometime after ~1300 years ago and was in use ~1100 years ago. These MAM-based ages come from two horizontal tube samples: one (KB14-05-1) obtained from sediments immediately underlying the clam garden wall (1.33 ± 0.15 ka), and the other (KB14-05-2) from sediments between clam garden wall rocks, 50 cm below the terrace surface (1.12 ± 0.15 ka) (Fig 2, Table 2).

**Fig 7:**
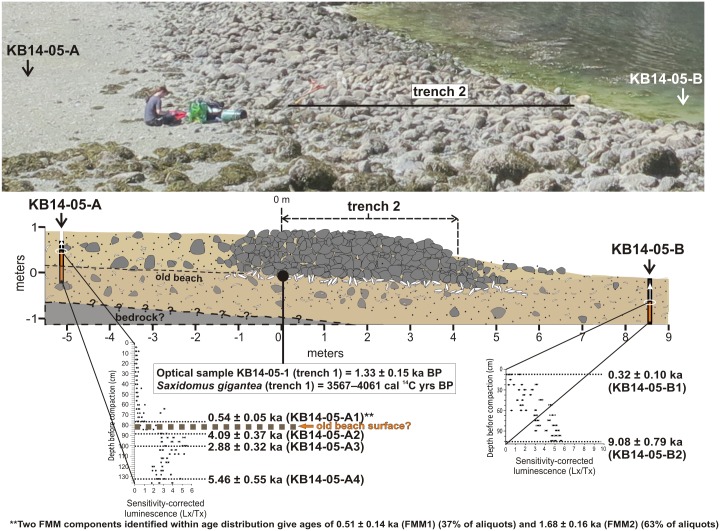
IRSL measurements and optical ages from core samples extracted near trench 2, site KB14-05. Due to compaction, the cores were only partially filled with sediment (brown shading) before extraction. Sensitivity-corrected IRSL measurements were made from subsamples collected every 2 cm along the length of the core. Age estimates were obtained above and below the horizon where the IRSL signal intensity increases dramatically from almost negligible levels in core KB14-05-A. This dramatic increase is interpreted to represent the pre-garden beach surface. Age estimates were also obtained from near the surface and near the base of core KB14-05-B. The ages of optical sample KB14-05-1 and a *S*. *gigantea* shell collected from below the wall in trench 1 are shown for comparison.

Further evidence that the wall was built within the last two millennia comes from an optical age from the base of the terrace infill behind the wall (core sample KB14-05-A1, Fig 7, Table 2). The D_e_ distribution of this sample has a high OD value (66%) that is largely a function of its bimodal distribution (Fig 6C and 6D). Because the bimodality of KB14-05-A1 may reflect sediment mixing of two sedimentary units (the pre-garden beach sands and overlying terrace fill), its D_e_ distribution was analyzed using the FMM. Two D_e_ components were identified (FMM1 and FMM2, Fig 6C and 6D). These give fading-corrected ages of 0.51 ± 0.14 ka (37% of aliquots, FMM1) and 1.68 ± 0.16 ka (63% of aliquots, FMM2) and OD values of 40%. We interpret these FMM component ages as evidence that the near-surface sand of the pre-garden beach was deposited ~1700 years ago (FMM2) and was overlain by sediment that infilled the terrace as a result of wall construction by at least ~500 years ago (FMM1), but probably earlier. Soon after sediment was deposited behind the wall, it would have been mixed by burrowing fauna and, we presume, human harvesting, which would have led to the exposure of sand grains to the sun (see *Evidence for incremental increase of wall height during construction*, below). Thus, we suspect that our FMM1 age approximates this most recent bleaching event and post-dates the time of terrace infilling. Terrace infilling probably commenced between ~1100 and ~1300 years ago as suggested by optical ages from sediments below, and within the clam garden wall (samples KB14-05-1 and KB14-05-2). The FMM2 component age from the pre-garden beach surface in core sample KB14-05-A1 is slightly older, but still overlaps with our below-wall optical age (1.33 ± 0.15 ka) within 2 sigma. If we calculate a MAM age from the aliquots in the FMM2 component (1.12 ± 0.13 ka), it is consistent within 2 sigma with the below-wall, and mid-wall optical ages obtained from the horizontal tube samples. The only radiocarbon age obtained from a *S*. *gigantean* shell from immediately below this wall was between 3567–4061 cal years BP. Again, this older age likely reflects relative sea level stability or slow regression during a ~3000 year period prior to wall construction that allowed for the accumulation and preservation of shells on the beach surface.

### Objective 2: Estimating the time and rate of sediment infilling behind clam garden walls

#### Evidence for increased sedimentation rates due to clam garden construction

Comparisons of the IRSL sensitivity-corrected signal intensities of the two vertical core samples from the Single Wall Site (KB14-05-A and KB14-05-B) suggest that the construction of clam gardens can increase sedimentation rates in the intertidal zone by at least fourfold (Fig 7). Vertical core KB14-05-B (seaward of the clam garden wall) shows a gradual increase in signal intensity from the beach surface down to a depth of 107 cm (before compaction), suggesting gradual and continuous sedimentation through time. Such a pattern in sedimentation would be expected for a beach that has not undergone periods of significant erosion or modification by humans. Two optical ages (KB14-05-B1 from the top of the core and KB14-05-B2 from the bottom of the core) suggest that ~1 m of sand (before compaction) was deposited on the beach during the last ~9000 years. This translates into a sedimentation rate of ~11 cm per thousand years.

The varied signal intensities in vertical core KB14-05-A (landward of the clam garden wall), however, reflects an abrupt change in sedimentation rate on the beach as a result of wall construction. This core shows very low signal intensities down to ~84 cm depth (before compaction), after which the signal intensity dramatically increases and remains bright down to ~136 cm (Fig 7). This pattern records the deposition of sediment infill (dim sensitivity-corrected signals) on the pre-garden beach sands (bright sensitivity-corrected signals) after construction of the clam garden wall. The dim signals of the sediment infill likely reflect relatively recent signal re-setting of the sands through bioturbation and mixing by humans during clam harvesting (see *Evidence for incremental increase of wall height during construction*, below).

We calculated the sedimentation rate of the pre-garden beach sand in core KB14-05-A using the FMM2 component age of sample KB14-05-A1 (1.68 ± 0.16 ka) that dates the near-surface pre-garden beach sediments, and the optical age of 5.46 ± 0.55 ka from pre-garden beach sediments near the base of core (sample KB14-05-A4) (Fig 7, Table 2). These ages suggest that the sedimentation rate of the pre-garden beach was only ~15 cm per thousand years, a value that is comparable to what we find on the non-walled beach in front of the wall (~11 cm per thousand years) and probably reflects deposition under natural conditions. If we assume that the clam garden wall was built shortly after ~1300 years ago (the MAM-based age of sample KB14-05-1, see Section *KB14-05 (Single Wall Site)*, above) and that terrace infilling commenced around this time and continues to the present day, the sedimentation rate of the terrace infill behind the clam garden wall would be ~58 cm per thousand years. Although over shorter timescales, infilling of the terrace was probably a spatially and temporally irregular process affected by rising and falling water levels and fluctuations in wave energy, our estimated sedimentation rate is approximately *four times* the sedimentation rate calculated for the pre-garden beach sands. This represents a dramatic rise that is the direct result of human modification and management of the landscape.

#### Evidence for incremental increase of wall height during construction

The IRSL signals measured from the length of core KB14-05-A suggest that the clam garden wall height at this site increased incrementally over time. Shell species on northern Quadra Island generally burrow into the substrate to depths of up to ~30–40 cm below the surface at any one time [18, 38], and mixing due to harvesting by humans (with digging sticks) are likely to be to a similar depth. However, because the optical signals in core KB14-05-A suggest that the entire ~84 cm thickness of the terrace infill has been mixed (i.e., the IRSL signal of most grains are relatively dim or show very slight increases with depth toward the pre-garden beach surface), infilling must have been gradual enough to allow mixing and bleaching of the lower-most terrace infill sediments (at depths greater than 40 cm) before the uppermost sediments were deposited. If the entire thickness of the infill was rapidly deposited shortly after wall construction, by contrast, we’d only see evidence of mixing in the form of recently bleached grains in the upper 40 cm of the terrace infill. Thus, our optical data support the notion that the clam garden wall was not built up to its present height in one, short-term construction event, but rather, incrementally over time, and possibly, over several generations as has been suggested by traditional knowledge holders.

## Discussion

Clam gardens, and indeed all rock-walled management features used by Indigenous people (e.g., fish traps and stone-faced plant gardens), are a fundamental component of the archaeological record and thus provide an avenue for understanding past resource and environmental management practices [39]. However, difficulties in establishing their age and determining how they developed through time, have stymied our ability to understand these practices. Optical dating techniques can provide ages for clam gardens when other options are not available. For instance, Lepofsky et al. [19] dated clam garden construction by radiocarbon dating barnacle basal plates (“barnacle scars”) on the underside of rocks at the base of garden walls, and shells at the interface between the pre-garden beach surface and terrace sediments. They found that while barnacle scars are an excellent way to date clam garden construction, their preservation requires very particular sedimentary and oceanographic conditions. Shells, which have been radiocarbon dated in the absence of barnacle scars, can survive intact on the surface of beaches for millennia, particularly during periods of relative sea level stability. Thus the temporal link between the shells and clam garden wall construction can be difficult to determine. Results of this study show that optical dating, and particularly a close examination of the IRSL signal intensities from vertical sediment cores, are invaluable for constraining the age of clam garden walls, especially in cases where barnacle scars or shells cannot be confidently associated with the time of wall construction. Given the uncertainty of dating clam gardens using conventional methods, optical dating will be the most efficient and economic means of determining the time of clam garden construction and use at many sites.

A disadvantage of optical dating is that sand grains in water lain sediments commonly contain a residual signal that can lead to high OD values and age overestimates. We have corrected in part for this by using the MAM in our age calculations (Table 3), but more definitive age estimates would be derived from single-grain D_e_ distributions [40]. Single-grain measurements would also confirm the presence of multiple age components, such as those that appear to exist in samples KB14-05-A1 and EbSh-58-1 that show congruencies between radiocarbon ages and field observations. Research into a time-efficient way of measuring fading rates from individual feldspar grains [41] or the properties of so-called post-IR IR signals from individual feldspar grains where fading is minimal or negligible [42] would be a step forward. Our measurements from quartz multi-grain aliquots suggest that most quartz grains are dim and lack the fast decay component desirable for dating (Supporting Information), however single-grain analysis might allow for the identification and dating of rare quartz grains that do not suffer from this problem [43]. Fading measurements, in this case, would not be necessary, and samples would contain fewer unbleached grains as the quartz luminescence signal resets more rapidly than that for Ksp. This approach may be impractical if suitable quartz grains are rare.

Nonetheless, our results show that optical dating in general can be used not only to determine *when* clam garden wall construction began, but also *how* clam gardens developed over time. Understanding how management features developed is a fundamental part of understanding the ethnoecology of traditional management practices. In the case of clam gardens, an outstanding research question is whether the clam garden walls were built to a particular tidal height in one construction event [18, 19], or whether they were built up slowly in an incremental fashion. Archaeological observations of the wall structure at KB14-05 and optical data from our vertical sediment core samples support the scenario that the clam garden wall and terrace were built up incrementally through time as people rolled rocks onto the walls, presumably while harvesting clams. This latter scenario reflects a future-focussed, continuous, and incrementally developing investment in these landscapes which is consistent with coastal First Nation traditional ecological knowledge. Future work that involves the extraction of vertical cores from other nearby clam gardens, such as the Double Wall Site where there is a distinct wall foundation underlying a less structured upper component, would allow us to test hypotheses concerning the timing of construction and whether the incremental growth of clam garden walls such as what we observed at the Single Wall Site (KB14-05) is consistent across features. Such data would further our understanding of Northwest coast marine resource management approaches and philosophies.

Our study uses optical dating of sediments for stone-walled features in which sediment terraces are integral to the function of the feature and where sediment is likely to accumulate in and landward of the wall. Similar sampling strategies that target abrupt changes in sedimentation rates may be used to constrain the age of other features such as rock-walled root gardens [44]. Although optical dating is typically conducted on sediments (sand or silt grains), in recent years, experiments have shown that rock surfaces may also yield reliable ages in certain geomorphic and archaeological contexts [45–49]. Therefore, an investigation into the feasibility of obtaining reliable optical ages from the surfaces of cobbles and boulders from stone structures is also underway. Dating rock surfaces at the base of clam garden walls should allow us to overcome problems in identifying and sampling pre-garden beach surfaces in the field. Rock surface dating techniques may also prove useful for determining the age of other structures such as fish traps that lack material suitable for radiocarbon dating [50]. We hope that by expanding our geochronological toolkit to include other stone-walled features and inorganic sample types such as boulders, we can refine our understanding of the complex history of human settlement and land use on the Pacific Northwest, and beyond.

## Supporting information

S1 Supporting Information (PDF)
