# Supplementary material for: Between a rock and a soft place: Using optical ages to date ancient clam gardens on the Pacific Northwest
Source: PLoS One. 2017 Feb 9;12(2):e0171775. doi: 10.1371/journal.pone.0171775 (PMC5300257; doi:10.1371/journal.pone.0171775)
Supplement: S1 Supporting Information — (PDF) [file pone.0171775.s001.pdf]

S1 Supporting Information

Environmental dose rate determination

The radiation absorbed by Ksp grains in the environment comes from alpha (α), beta (β) and gamma (γ) radiation emitted during the decay of U, Th, K and Rb within the grains and from their surrounding sediments, and from cosmic rays originating from space. In this study, the effects of α radiation on the Ksp grains has been removed by etching their surfaces using HF. U, Th, K and Rb parent contents were measured from the outermost (light-exposed) sediment extracted from the end of each tube sample, and from each core subsample used for optical dating using neutron activation analysis (NAA) (Table A). This method does not account for spatial heterogeneities in the sediment matrix that may affect the γ dose rate at the sample site; for example the presence of a pebble or boulder near (within ~ 50 cm) of the sample site may contribute more to the γ ray dose absorbed by a sample than the surrounding shell-rich sediment. Thus, three subsamples of one granodiorite boulder (typical of the lithology of clasts at the sample sites) that was extracted from the base of the wall at site KB14-05 (trench 2), were also milled and analyzed for radionuclide concentration. Our measured γ dose rates from the granodiorite samples are ~38-190% higher than those measured from our sediment samples. A γ ray flux model by Aitken [1] indicates that inert soil will absorb ~22% of the γ dose emitted by a high-dose boulder only 5 cm away (his table H.1 and figure H.1). But if we assume that sample KB14-05-2 was this close to the granodiorite boulder, the dose rate of KB14-05-2 would increase by only 4.5% (to 1.87 ± 0.16 Gy/ka) and the new MAM age (1.07 ± 0.14 ka) would remain consistent within 1 sigma with the value in Table 3. This suggests that spatial heterogeneities in γ dose rates at our samples sites should not lead to significant age underestimations; more accurate environmental dose determinations will require either *in situ* γ spectrometry measurements in the field or modelling of the spatial distribution of low-dose and high-dose components at each sample site [2].

Dose rates were calculated assuming secular equilibrium in the decay chains. It should be noted that all samples have been obtained from beach sands in an intertidal environment and it is possible that U has migrated in or out of the sands since they were originally deposited. If the current state of the U decay chain differs significantly from that in the past, then using the U contents measured at the time of sample collection in the dose rate calculations may yield inaccurate results [3, 4]. This issue has been shown to be minor in well drained sand [4, 5] such as that found at the sites in this study.

Table A. Dose rate information[1].

Sample (Laboratory ID)	Total dose rate (Gy/ka)	Sample depth[4] (cm)	Water content[5] (Δ^w)	Rb (ppm)	Th (ppm)	U (ppm)	K (%)
KB14-05-1 (KB1405c)	1.46 ± 0.10	100	0.363 ± 0.030	9.00 ± 2.07	0.50 ± 0.05	0.80 ± 0.17	0.30 ± 0.10
KB14-05-1[2] (KB1405c)	1.43 ± 0.08	100	0.363 ± 0.030	8.6 ± 0.1	0.434 ± 0.023	0.71 ± 0.08	0.23 ± 0.02
KB14-05-2 (Quad10)	1.78 ± 0.16	72	0.314 ± 0.031	15.00 ± 1.40	1.20 ± 0.07	<0.7	0.80 ± 0.09
EbSh-5-1 (KB07)	1.88 ± 0.13	90	0.277 ± 0.028	18.00 ± 2.52	1.30 ± 0.08	0.80 ± 0.19	0.80 ± 0.10
EbSh-58-1 (TWB01)	1.91 ± 0.12	30	0.213 ± 0.021	17.00 ± 2.89	1.80 ± 0.11	0.50 ± 0.05	0.80 ± 0.08
KB14-05-A1 (Quad5u)	1.90 ± 0.13	76	0.363 ± 0.030	17.00 ± 1.60	1.80 ± 0.11	0.90 ± 0.30	0.90 ± 0.10
KB14-05-A2 (Quad5x)	2.07 ± 0.15	88	0.363 ± 0.030	16.00 ± 1.40	1.60 ± 0.09	1.20 ± 0.30	1.10 ± 0.11
KB14-05-A3 (Quad5z)	1.78 ± 0.12	100	0.363 ± 0.030	17.00 ± 2.89	1.30 ± 0.08	0.60 ± 0.13	0.80 ± 0.10
KB14-05-A4 (Quad5z8)	1.78 ± 0.12	133	0.363 ± 0.030	21.00 ± 2.80	1.40 ± 0.09	0.50 ± 0.13	0.80 ± 0.10
KB14-05-B1 (Quad9-16)	1.90 ± 0.12	8	0.363 ± 0.030	17.00 ± 1.50	1.10 ± 0.07	<0.7	1.00 ± 0.10
KB14-05-B2 (Quad9-03)	1.82 ± 0.13	105	0.363 ± 0.030	17.00 ± 1.50	1.10 ± 0.06	0.90 ± 0.30	0.80 ± 0.09
Crushed granodiorite[3]	2.37 ± 0.09	100	0	26.1 ± 1.0	1.67 ± 0.05	0.89 ± 0.07	0.95 ± 0.06

[1] Rb, U, Th and U concentrations were determined using neutron activation analysis (NAA) at Maxxam Analytics. The internal (inside the grain) dose rate contributions from K and Rb, and the external (outside the grain) contributions of all radionuclides was calculated for 180–250 μm diameter grains using the conversion factors of Guérin et al. [6] and attenuation and absorption coefficients from Guérin et al. [7]. These have been adjusted to account for etching with HF acid using corrections by Nathan [8]. The α contribution from inside Ksp grains was assumed to be 0.09 Gy/ka based on Ollerhead et al. [9]. Because water in the sediments attenuates β and γ radiation, dose rates of all samples were corrected for water content.

[2] Rb, U, and Th concentrations were determined using NAA, and the U was analyzed using delayed neutron counting at the Australian Nuclear Science and Technology Organisation (ANSTO), as this method can detect lower concentrations of U.

[3] Weighted mean values from three crushed subsamples of one granodiorite boulder extracted from the base of the clam garden wall at site KB14-05 (trench 2).

[4] Depths of samples KB14-05-A1, KB14-05-A2, KB14-05-A3, KB14-05-A4, KB14-05-B1 and KB14-05-B2 have been estimated graphically, assuming that all extracted sediment (including what was lost during extraction) filled the entire vertical core hole before compaction during sampling.

[5] Water contents are derived from samples that were saturated in the laboratory. The water content of sample KB14-05-1 was used for core samples KB14-05-A1, KB14-05-A2, KB14-05-A3, KB14-05-A4, KB14-05-B1 and KB14-05-B2 from the same clam garden.

Preparation of samples for optical dating

The 180–250 μm diameter grain-size fraction of all samples used for optical dating was treated with HCl acid (10%) and H_2O_2 acid (10%) to remove any traces of carbonates and organic material, and Ksp and quartz grains were concentrated using heavy liquid. Both minerals were treated with HF acid (50% for 45 minutes for quartz and 10% for 5 minutes for Ksp) to remove the outermost part of the grains that would have been exposed to α radiation. Ksp and quartz grain concentrations were then mounted separately on aluminum discs using silicone oil and a 2 mm mask (equal to 99 ± 21

grains per aliquot as counted from 17 aliquots) to ensure adequate signal intensities. All optical stimulations and irradiations were performed on a Risø TL/OSL DA-20 reader [10] equipped with a calibrated $^{90}Sr/^{90}Y$ β source that delivered β particles to the sample at a rate of ~5–6 Gy/min. The luminescence signal from Ksp in all samples extracted from the cores, as well as the samples used for optical dating was measured at 50 ºC using infrared (IR) (880 nm) light. The blue-violet (~400 nm) emissions were detected using an Electron Tubes Ltd. 9235QA photomultiplier tube fitted with Schott BG-39 and Corning 7-59 filters. The signal from quartz was measured at 125 ºC using blue (470 nm) light and ultraviolet (~350 nm) emissions were detected using the same photomultiplier tube but fitted with a 7.5 mm thick Hoya U-340 optical filter. The equivalent dose (D_e) values of each Ksp multi-grain aliquot was determined using a single-aliquot regenerative-dose (SAR) procedure (see below) and up to 45 aliquots were measured per sample.

Determining the suitability of Quadra Island quartz and Ksp for optical dating

Sands found in clam gardens and in beaches on the coasts of Quadra Island are largely derived from glacigenic sediments deposited during the last (Fraser) glaciation (i.e., glaciofluvial outwash and till) that have been re-worked by fluvial and littoral processes during postglacial time [11]. The sands are typically dominated by plagioclase feldspar and quartz with lesser amounts of other minerals that have been eroded from plutonic rocks (quartz monzodiorites and diorites, tonalities and granodiorites) cropping out along the southern coast of British Columbia [11]. Quartz continuous wave optically-stimulated luminescence (CW-OSL) signals, measured using steady stimulation power, are dim. Linearly-modulated (LM-) OSL curves, measured while gradually increasing the stimulation power, indicate that the quartz lacks the thermally stable fast component which is desirable for dating [12] (Fig A). This is probably because the quartz in these sands has experienced relatively few cycles of erosion and re-deposition (burial) which would have resulted in repeated bleaching and irradiation of the grains. This is known to increase the intensity of luminescence per dose of irradiation (sensitivity) of the quartz [13, 14]. Thus all optical samples in this study have been dated using Ksp.

Equivalent dose measurements

The D_e values for Ksp multi-grain aliquots were determined using the SAR measurement procedure shown in Table B. This procedure involved measurement of the 'natural' IRSL signal (L_n), followed by measurement of the corresponding signal induced by a test dose of ~1 Gy given in the laboratory (T_n). A dose-response curve was then generated from the luminescence signals induced by a series of regenerative doses given in the laboratory (L_x), each of which was followed by measurement of the test dose signal (T_x) to correct for any sensitivity changes [15, 16]. A regenerative dose of 0 Gy

A

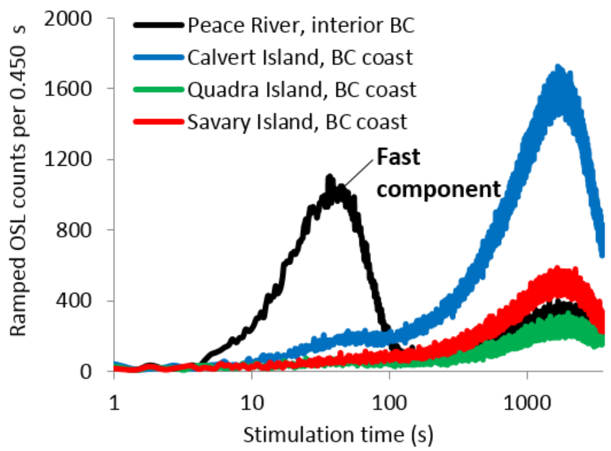

B

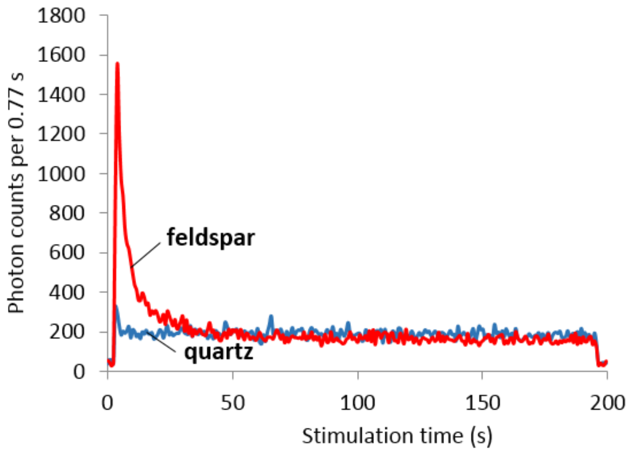

Fig A. A) Linearly-modulated optically stimulated luminescence (LM-OSL) signals from quartz from BC's coast (including Quadra Island) and the interior of British Columbia (Peace River). Quartz from the coast lacks the fast component required for dating. B) Continuous wave optically stimulated luminescence (CW-OSL) signals from quartz and Ksp from sample KB14-05-1.

was also given as part of the SAR sequence, and the 'zero-dose' signal measured to check for any recuperation (the thermal or optical transfer of charge into the IR-sensitive traps measured for dating). A repeat regenerative dose of 68 Gy was given to each aliquot after the zero-dose cycle, and the induced signal measured to check that any sensitivity changes had been taken into account; the 'recycling ratio' ('double-regenerative' ratio of Galbraith et al. [15]) should be close to unity if the sensitivity correction has been successful. The resulting sensitivity-corrected dose-response curve of L_x/T_x ratios was fitted with a single saturating exponential function of the form:

$$I = I_0 + I_{max}(1 - e^{-\frac{D}{D_0}}) \qquad \text{(A)}$$

where I is the sensitivity-corrected luminescence signal, I_0 and I_{max} refer to the signals measured at the beginning and the end of the dose-response curve, respectively (both estimated parameters in the fitted curve), D is the given laboratory dose, and D_0 is a constant (the characteristic saturation dose) that describes the shape of the curve. The D_e for each aliquot was estimated by interpolation by projecting L_n/T_n on to the dose-response curve. For each L_n, T_n, L_x and T_x, measurement was made for a stimulation duration of 200 s, and the signal and background count rates were determined from the first 1 s and the last 20 s of IR stimulation, respectively. In addition to photon counting statistics, an instrumental error of 1.5% was added in quadrature to each of these measurements, following Duller [17]. The D_e was calculated using Analyst version 3.24, and the standard error estimated by Monte Carlo stimulation [17]. The luminescence characteristics (dose response curve shape, recuperation and recycling ratio values) and D_e distributions of all samples are shown in Fig B. As expected, younger samples with dimmer signals yielded more scatter in the recuperation values and recycling ratios, which in turn, led to higher aliquot rejection rates (e.g., only 22 out of 71 accepted aliquots for sample KB14-05-B1).

KB14-05-1

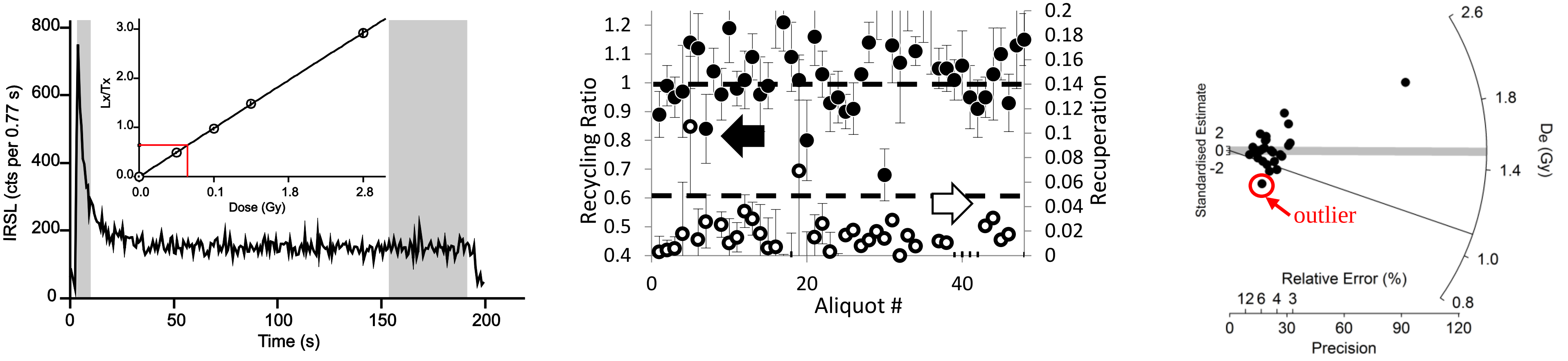

KB14-05-2

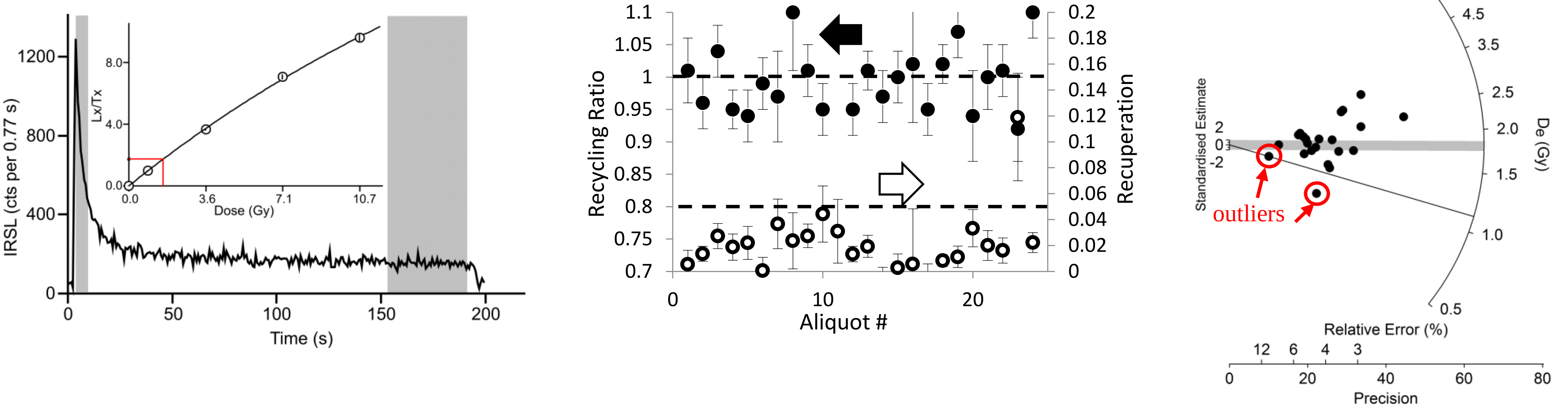

Fig B. Typical luminescence decay curves, dose response curves (inset graphs), recuperation, recycling ratio and equivalent dose (D_e) data for all samples measured. Grey shading on the decay curves marks the initial and background signal integrals used for D_e calculation. Grey shading in the radial plots marks ±2σ of the CAM D_e value, and the solid lines mark the MAM D_e value calculated after excluding lowest-most outliers. FMM component D_e values are shown as red dashed lines in the radial plots of samples EbSh-58-1 and KB14-05-A1 (see text for explanation). Only aliquots which pass all rejection criteria are plotted in the radial plots. Because the D_e values of sample KB14-05-B1 are so small, its D_e distribution is plotted on a linear scale. The distributions of all others are plotted on a log scale.

EbSh-5-1

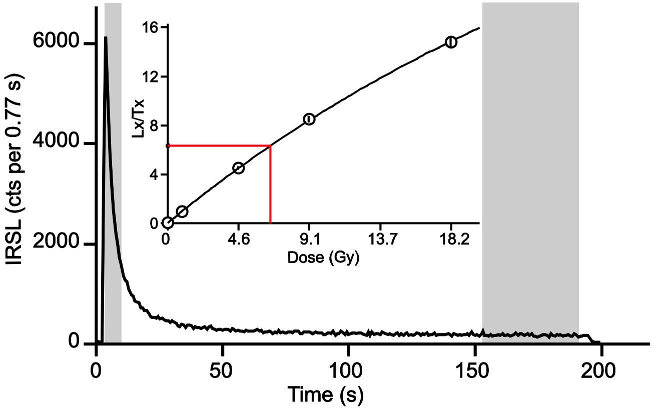

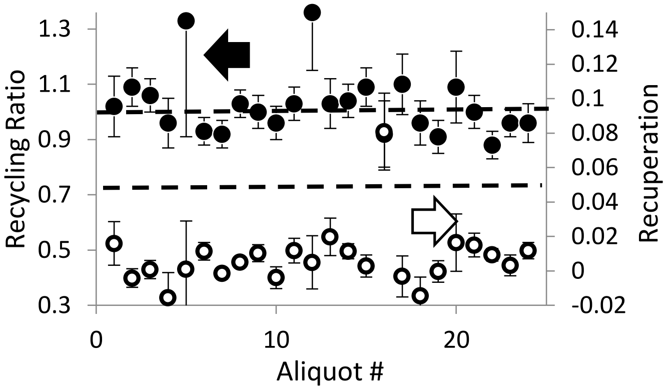

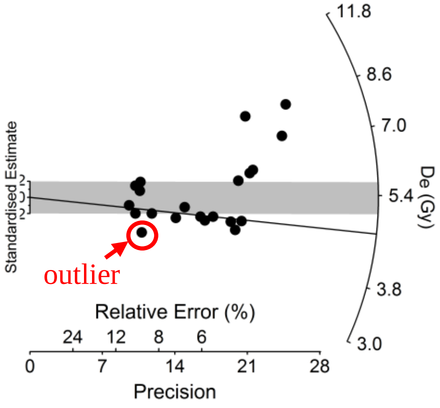

EbSh-58-1

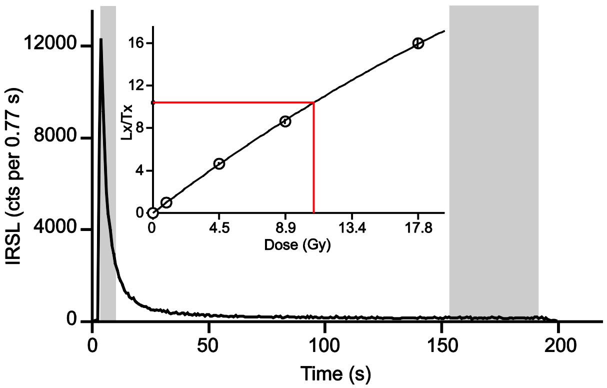

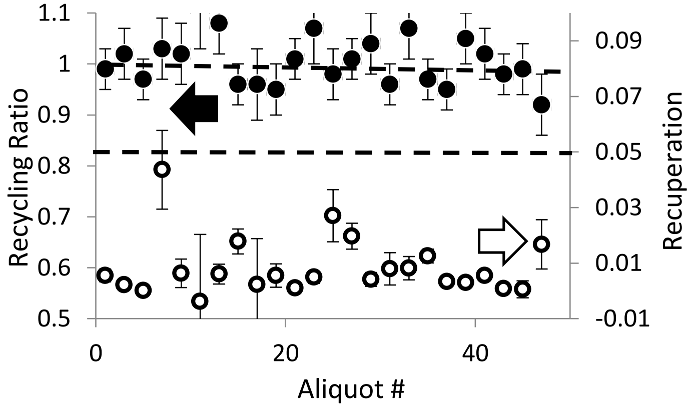

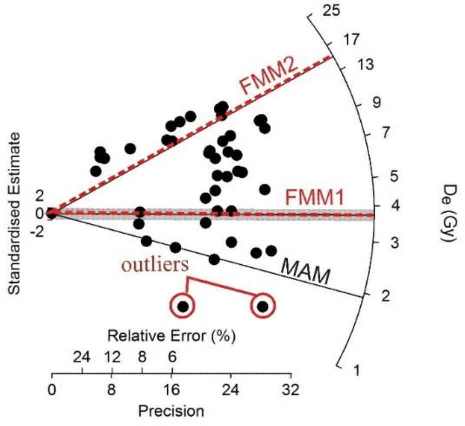

Fig B continued…

KB14-05-A1

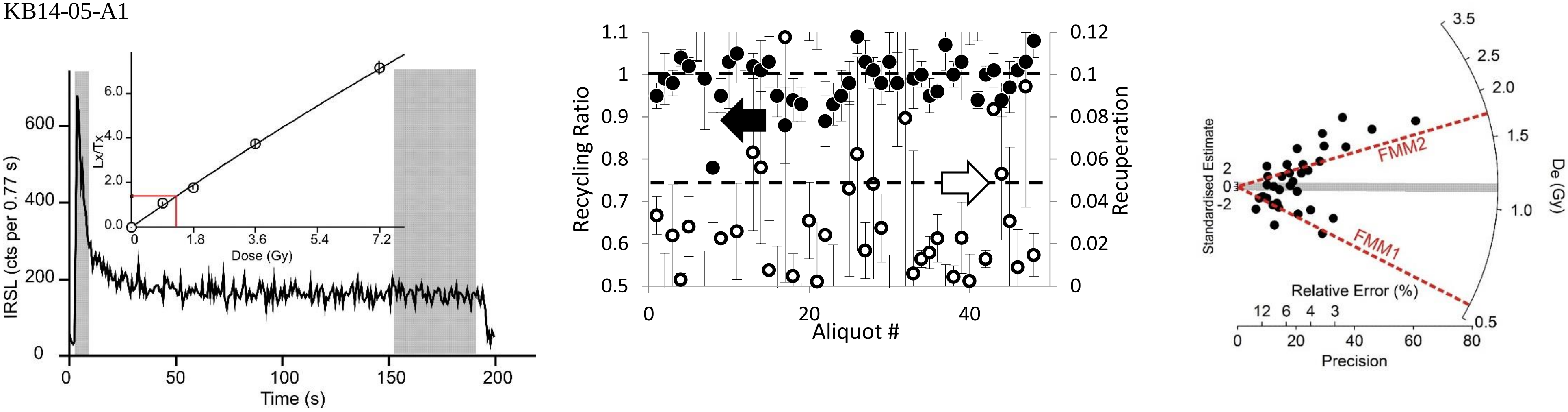

KB14-05-A2

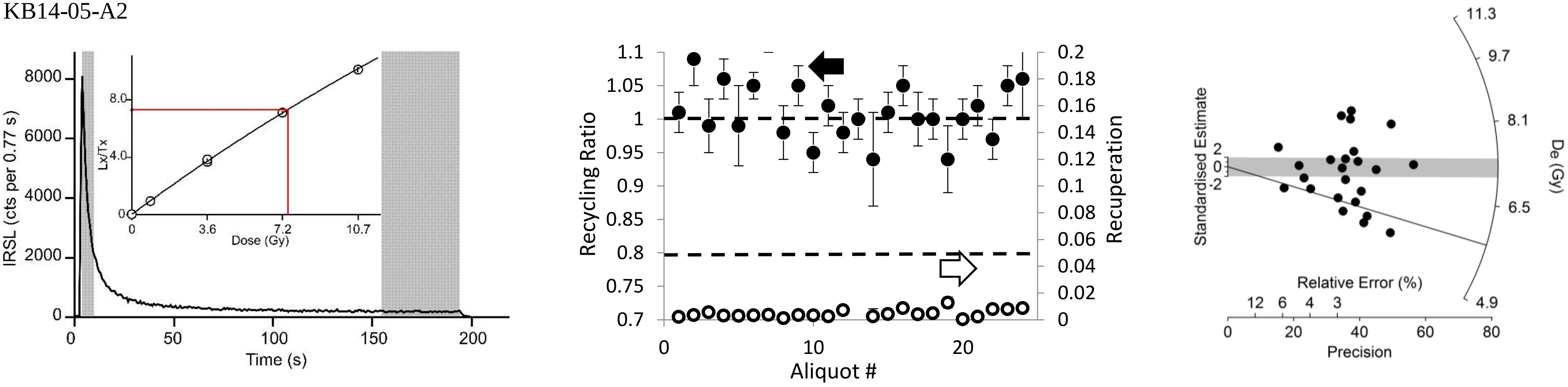

Fig B continued…

KB14-05-A3

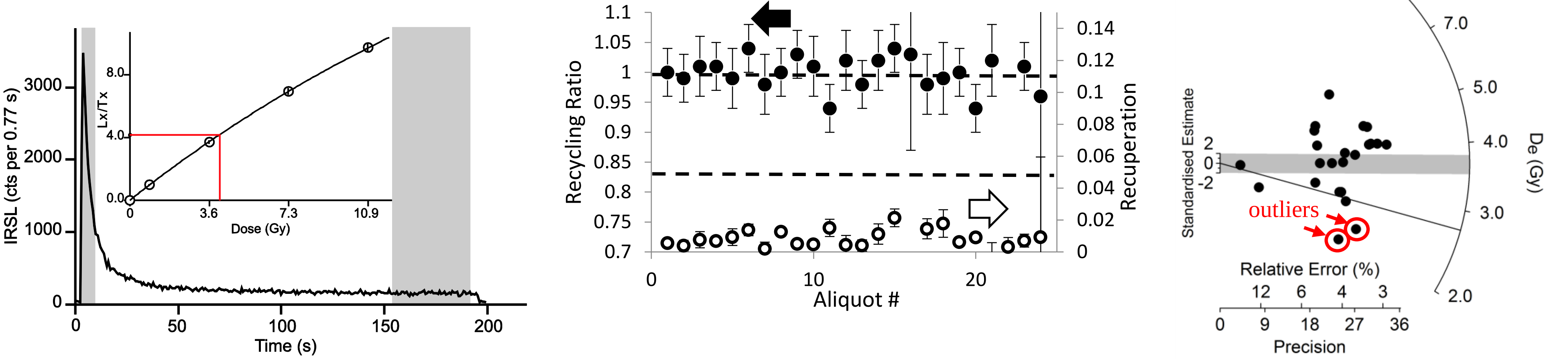

KB14-05-A4

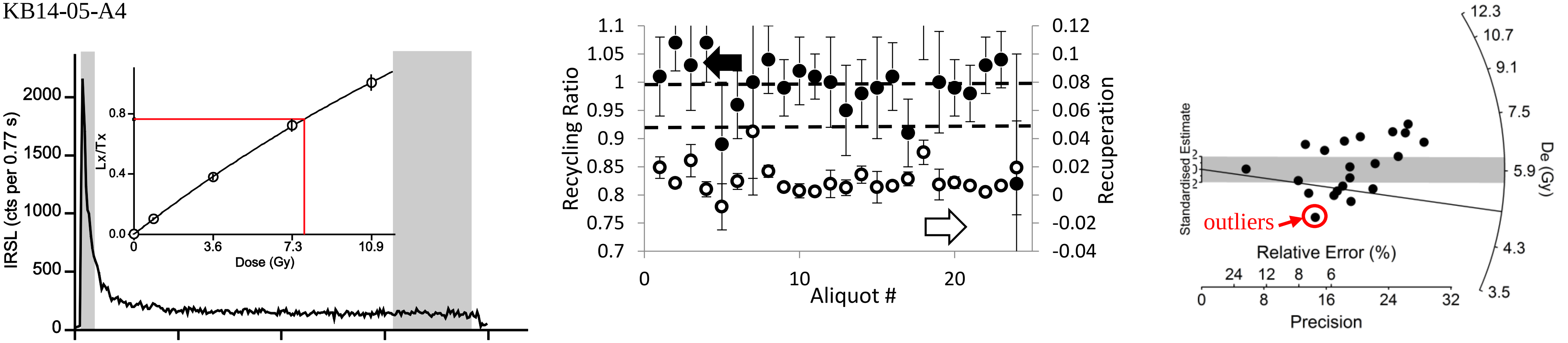

Fig B continued…

KB14-05-B1

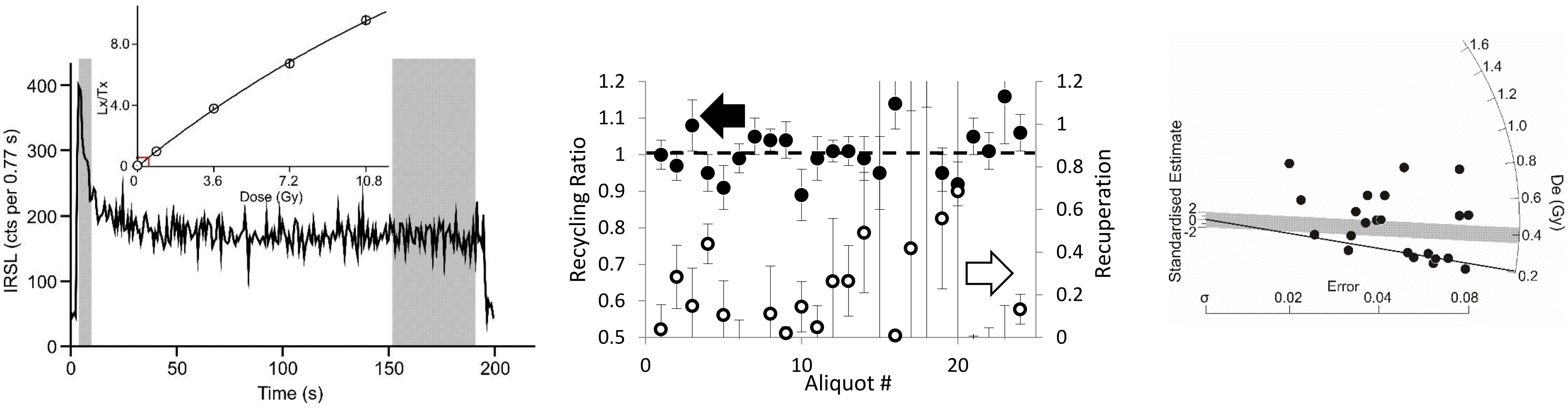

KB14-05-B2

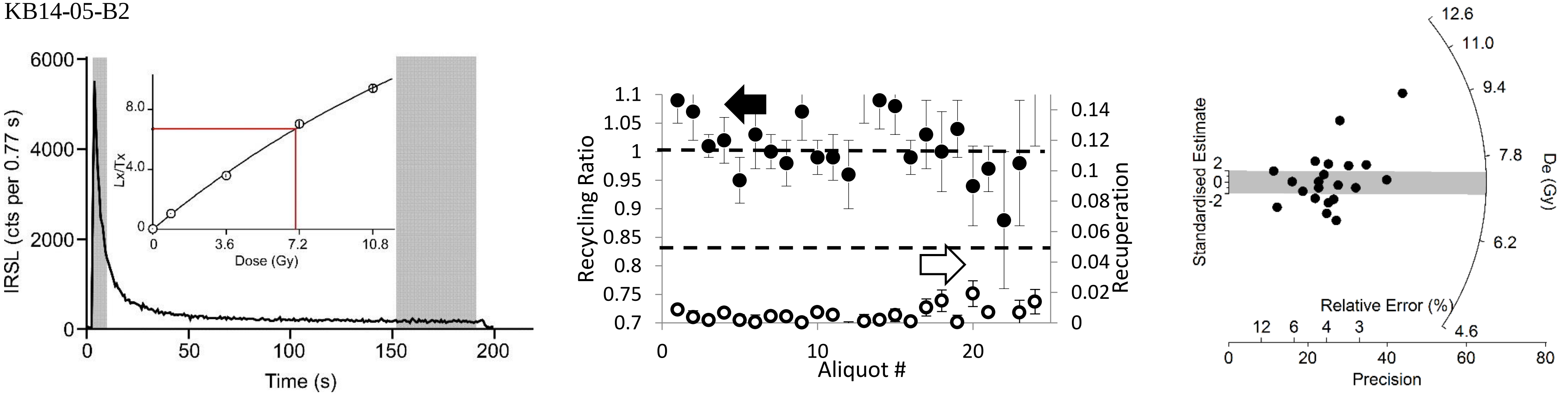

Fig B continued…

Table B. SAR protocol for Ksp grains used in this study.

1.	Natural / Regenerative Dose[1]
2.	Preheat (160°C, 10 s)
3.	IRSL (50°C, 200 s) → L_n, L_x
4.	Test dose (0.94 Gy)
5.	Preheat (160°C, 10 s)
6.	IRSL (50°C, 200 s)….→ T_n, T_x
8.	Hotwash (180°C, 40 s)[2]
9.	Return to step 1.

[1] Ln = natural signal, Lx = regenerative dose signal. A "zero dose" point was measured before the second highest regenerative dose to measure recuperation and build-up of background signal, and a repeat dose point was measured after the highest regenerative dose for the recycling ratio.

[2] A hotwash temperature that is higher than the preheat temperature is included in the sequence to reduce recuperation, following Murray and Wintle [18].

Dose recovery test

In order to see if our SAR protocol was suitable for Ksp grains on Quadra Island, a dose recovery test [19] was conducted on 24 aliquots of Ksp from sample KB14-05-1. Because our samples are from water lain deposits that were likely exposed to less shortwave radiation (UV) than wind-transported sand, we bleached our grains using IR diodes rather than exposing them to direct sunlight, as is commonly done, before giving them a known dose of radiation in the laboratory. Shortwave radiation can cause unwanted phototransfer of charge to the traps being sampled during measurement either from other traps, or directly from the valence band [20]. This phototransfer may lead to over-estimates of the D_e, particularly in young samples. Aliquots were rejected from analysis if their recuperation value was equal to or greater than 5% of the natural signal and if their recycling ratio deviated from unity by more than 10% [21]. Only one aliquot failed to pass the rejection criteria in the dose recovery test and the measured-to-given dose ratio was 1.00 ± 0.01 with an OD of 0% (Fig C). Thus we concluded that our SAR protocol is suitable for dating Ksp on Quadra Island.

Before calculating ages using the MAM, we removed any low outliers from the D_e distribution (Fig B) and added (in quadrature) OD values of 10% to each of the D_e measurement errors to allow for unexplained scatter commonly observed in well-bleached deposits [22, 23]. The value of 10% is the maximum expected as the OD value of our dose recovery test result (simulating complete depletion of the optical signal in sands in a subaqueous environment) was 0%.

A

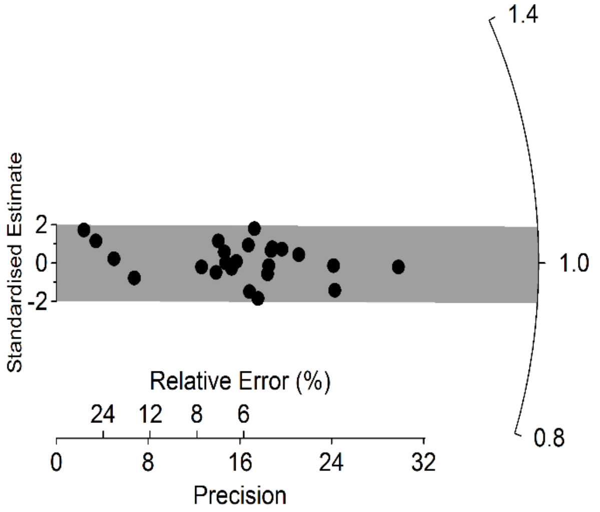

B

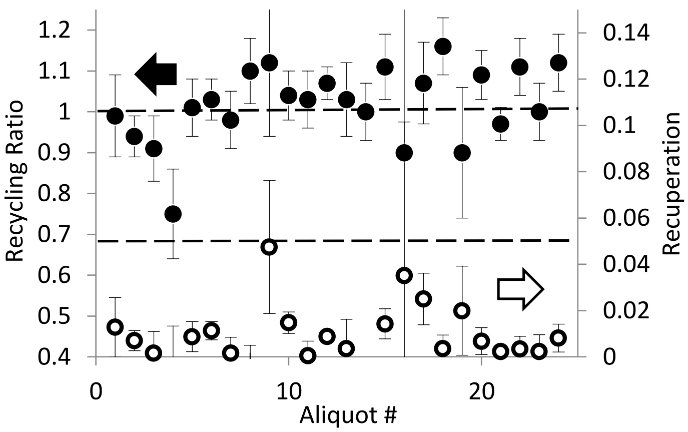

Fig C. A) Dose recovery test results (radial plot). The sample was bleached in the machine using IR diodes to prevent phototransfer effects from sunlight. B) Recycling ratios and recuperation values for all aliquots. Aliquots with ideal characteristics have recuperation values <0.05 and recycling ratios between 0.9 and 1.1.

Anomalous fading measurements

The thermally stable IRSL luminescence signal from feldspar is well known to fade over time (a process known as anomalous fading) [24]. To correct for this effect, anomalous fading rates were measured from seven samples (KB14-05-1, KB14-05-2, KB14-05-A1, KB14-05-A2, KB14-05-B1, EbSh-5-1, EbSh-58-1) using the SAR measurement procedure of Auclair et al. [25]. All fading rates (*g*) were normalized to a delay period of 2 days [24]. As expected, there is inter-aliquot variability in fading rates, and sample-averaged fading rates are similar to those of samples measured from the central coast of BC, ranging from ~4.2 to ~8.0 %/decade [21] (Fig D). All optical ages were corrected for fading using the model of Huntley and Lamothe [24].

FMM calculations

Justification for applying the FMM to the multi-aliquot D_e distributions of samples EbSh-58-1 and KB14-05-A1

In this study, we apply the FMM to the multi-grain aliquot D_e distributions of two samples that show evidence of sediment mixing (EbSh-58-1 and KB14-05-A1). Arnold and Roberts [26] caution against the use of the FMM on multi-grain aliquot D_e distributions as their simulations have shown that, even with small (25 grain) aliquots, spurious "phantom" components may be generated that are not part of the original single-grain mixture. These phantom components represent aliquots with a mixture of grains from more than one component, and commonly occur between two components (measured from multi-grain aliquots) that are statistically equivalent in age to the true components of the single-grain mixture (e.g., their figure 2a-c and table 2). However, because the D_e distributions of samples EbSh-58-1 and KB14-05-A1 have no more than two FMM components in total, and the ages calculated from these components are consistent with what we expect for these sites, we suspect that these reflect the ages of two 'real' populations of grains (i.e., the pre-garden beach sands and postglacial outwash in the case of EbSh-58-1, and pre-garden beach sands and the younger sediment infill in the case of KB14-05-A1). Arnold and Roberts' simulations show that when a single-grain mixture is comprised of grains from two different age populations, the FMM almost always detects the age of these two components when applied to multi-grain aliquots of the same sample (their table 2), supporting our interpretation. Ideally, optical dating at the single grain level is required to confirm the existence of two components in our samples, but unfortunately for feldspars this would require determining the fading rates of individual grains [27] and this is too impractical to be routine practice.

A B

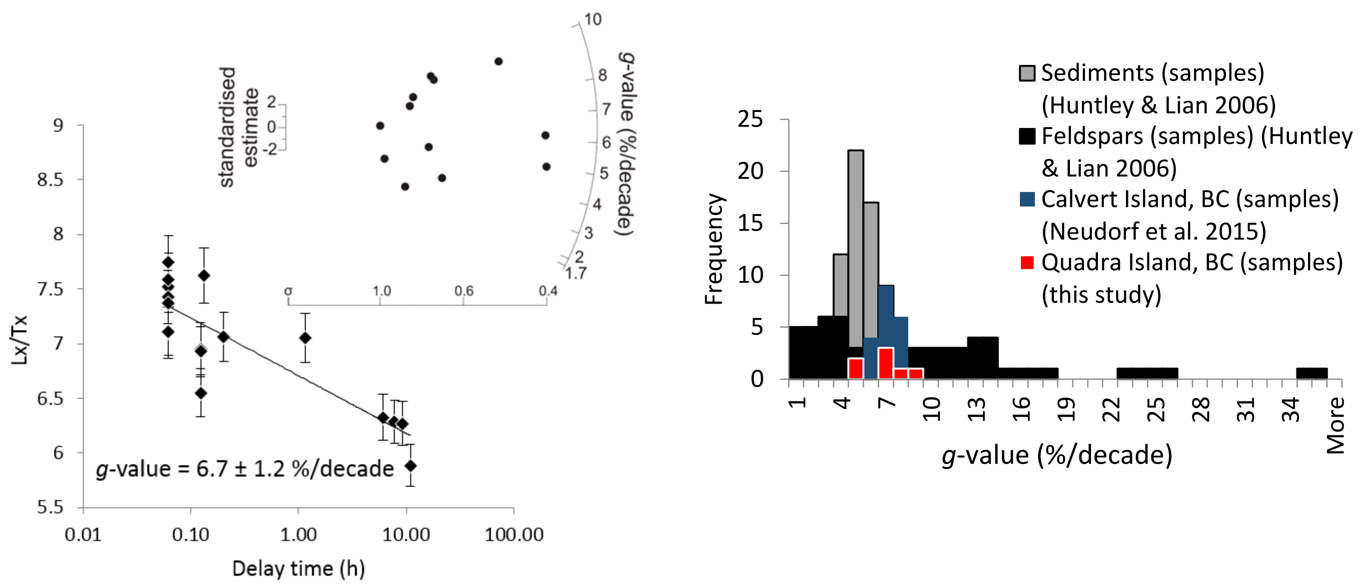

Fig D. A) Fading plot for one aliquot from sample KB14-05-1. The *g*-value distribution for all 12 aliquots measured from this sample are shown in the inset radial plot. B) Weighted mean fading rates of all Quadra Island samples plotted with fading rates of sediments from Calvert Island, BC central coast as well as feldspars from various locations in Canada and overseas.

Investigation of the possible effects of inter-aliquot variability in fading rates on FMM calculations

Because fading tests show that fading rates vary from aliquot to aliquot (Fig D), we conducted an experiment on sample KB14-05-A1 to see if the same number of components would be identified from the distribution after correction using aliquot-specific fading rates. Corrections were applied two ways: i) assuming all aliquots have the same fading rate (6.82 ± 0.16 %/decade, the weighted mean *g*-value of 12 aliquots) as in Table 3, and ii) assuming aliquots have unique fading rates (randomly generated *g*-values ranging from 3.7 %/decade and 13.3 %/decade, the maximum and minimum values measured from 12 aliquots of sample KB14-05-A1) (Fig E). Corrections using the second method were applied five times using five different sets of randomly generated *g*-values. In all cases, two FMM components best fit the data, and these had OD values ranging from 30 to 60% (Table C, Fig E). The fading-corrected ages of FMM1 and FMM2 that assume all aliquots have the same fading rate are statistically consistent (within 2 sigma) with the corresponding components in all calculations that assume aliquot-specific fading rates (iterations 1-5, Table C). These results suggest that the aliquot-to-aliquot variations in fading rate that likely exist within our samples are probably not sufficient to obscure the detection of any FMM components that differ in age by ~1000 years or more.

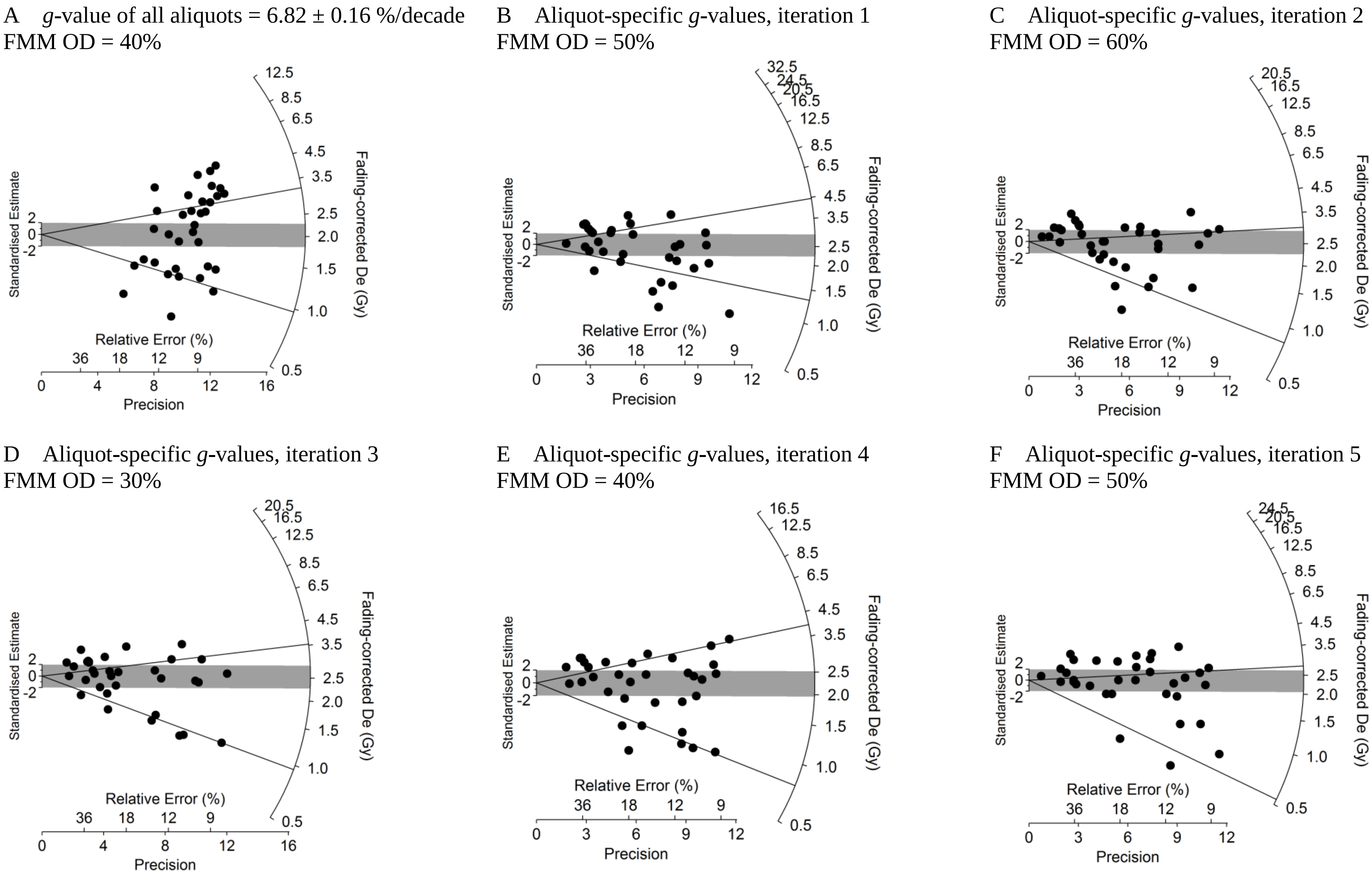

Fig E. A) The D_e distribution of sample KB14-05-A1, assuming all aliquots have a fading rate of 6.82 ± 0.16 %/decade (the fading rate of this sample). B-F) Fading-corrected D_e distributions of sample KB14-05-A1 assuming all aliquots have a unique fading rate. Corrections were applied five times using randomly generated *g*-values within the range of those measured from 12 aliquots of KB14-05-A1 (i.e., between 3.7 %/decade and 13.3 %/decade). The shaded region is ± 2σ of the CAM weighted mean and FMM component D_e values are shown as solid black lines in all plots. The FMM OD is the OD that best fits two components identified by the FMM model.

Table C. FMM component ages for sample KB14-05-A1 after fading correction. See Table A for dose rate information.

	g-value of all aliquots = 6.82 ± 0.16 %/decade	Aliquot-specific *g*-values, iteration 1	Aliquot-specific *g*-values, iteration 2	Aliquot-specific *g*-values, iteration 3	Aliquot-specific *g*-values, iteration 4	Aliquot-specific *g*-values, iteration 5
FMM1 age	0.51 ± 0.14 ka	0.70 ± 0.14 ka	0.44 ± 0.47 ka	0.51 ± 0.08 ka	0.42 ± 0.11 ka	0.27 ± 0.23 ka
FMM2 age	1.68 ± 0.16 ka	2.35 ± 0.21 ka	1.58 ± 0.16 ka	1.88 ± 0.14 ka	1.64 ± 0.13 ka	1.47 ± 0.12 ka

References

1. Aitken, MJ. Thermoluminescence Dating. London: Academic Press; 1985.
2. Martin, L, Icerti, S, Mercier, N. DosiVox: Implementing Geant 4-based software for dosimetry simulations relevant to luminescence and ESR dating techniques. Ancient TL. 2015; 33: 1-10.
3. Lian, OB, Hu, J, Huntley, DJ, Hicock, SR. Optical dating studies of Quaternary organic-rich sediments from southwestern British Columbia and northwestern Washington State. Can J Earth Sci. 1995; 32: 1194-1207.
4. Olley, JM, Murray, AS, Roberts, RG. The effects of disequilibria in the uranium and thorium decay chains on burial dose rates in fluvial sediments. Quaternary Sci Rev. 1996; 15: 751-760.
5. Prescott, JR, Hutton, JT. Environmental dose rates and radioactive disequilibrium from some Australian luminescence dating sites. Quaternary Sci Rev. 1995; 14: 439-448.
6. Guérin, G, Mercier, N, Adamiec, G. Dose rate conversion factors: update. Ancient TL. 2011; 29: 5-8.
7. Guérin, G, Mercier, N, Nathan, R, Adamiec, G, Lefrais, Y. On the use of the infinite matrix assumption and associated concepts: A critical review. Radiat Meas. 2012; 47: 778-785.
8. Nathan, RP. Numerical modelling of the environmental dose rate for trapped-charge dating. PhD Thesis, University of Oxford. 2010.
9. Ollerhead, J, Huntley, DJ, Berger, GW. Luminescence dating of sediments from Buctouche Spit, New Brunswick. Can J Earth Sci. 1994; 31: 523-531.
10. Bøtter-Jensen, L, Thomsen, KJ, Jain, M. Review of optically stimulated luminescence (OSL) instrumental developments for retrospective dosimetry. Radiat Meas. 2010; 45: 253-257.
11. Cummings, JM. Preliminary investigation into possibilities for producing silica sand from British Columbia sand deposits. Victoria: British Columbia Department of Mines; 1941.
12. Jain, M, Murray, AS, Bøtter-Jensen, L. Characterisation of blue-light stimulated luminescence components in different quartz samples: implications for dose measurement. Radiat Meas. 2003; 37: 441-449.
13. Pietch, TJ, Olley, JM, Nanson, GC. Fluvial transport as a natural luminescence sensitiser of quartz. Quat Geochronol. 2008; 3: 365-376.
14. Jeong, GY, Choi, J- H. Variations in quartz OSL components with lithology, weathering, and transportation. Quat Geochronol. 2012; 10: 320-326.
15. Galbraith, RF, Roberts, RG, Laslett, GM, Yoshida, H, Olley, JM. Optical dating of single and multiple grains of quartz from Jinmium rock shelter, northern Australia: Part I, experimental design and statistical models. Archaeometry. 1999; 41: 339-364.
16. Murray, AS, Wintle, AG. Luminescence dating of quartz using an improved single-aliquot regenerative-dose protocol. Radiat Meas. 2000; 32: 57-73.
17. Duller, GAT. Assessing the error on equivalent dose estimates derived from single aliquot regenerative dose measurements. Ancient TL. 2007; 25: 15-24.
18. Murray, AS, Wintle, AG. The single-aliquot regenerative dose protocol: potential for improvements in reliability. Radiat Meas. 2003; 37: 377-381.
19. Roberts, RG, Galbraith, RF, Olley, JM, Yoshida, H, Laslett, GM. Optical dating of single and multiple grains of quartz from Jinmium Rock shelter, northern Australia: Part II, Results and implications. Archaeometry. 1999; 41: 365-395.
20. Huntley, DJ, Clague, JJ. Optical Dating of Tsunami-Laid Sands. Quaternary Res. 1996; 46: 127-140.
21. Neudorf, CM, Lian, OB, Walker, IJ, Shugar, DH, Eamer, JBR, Griffin, LCM. Toward a luminescence chronology for coastal dune and beach deposits on Calvert Island, British Columbia central coast, Canada. Quat Geochronol. 2015; 30: 275-281.
22. Galbraith, RF, Roberts, RG, Yoshida, H. Error variation in OSL palaeodose estimates from single aliquots of quartz: a factorial experiment. Radiat Meas. 2005; 39: 289-307.

23. Galbraith, RF, Roberts, RG. Statistical aspects of equivalent dose and error calculation and display in OSL dating: an overview and some recommendations. Quat Geochronol. 2012; 11: 1-27.

24. Huntley, DJ, Lamothe, M. Ubiquity of anomalous fading in K-feldspars and the measurement and correction for it in optical dating. Can J Earth Sci. 2001; 38: 1093-1106.

25. Auclair, M, Lamothe, M, Huot, S. Measurement of anomalous fading for feldspar IRSL using SAR. Radiat Meas. 2003; 37: 487-492.

26. Arnold, LJ, Roberts, RG. Stochastic modelling of multi-grain equivalent dose (De) distributions: Implications for OSL dating of sediment mixtures. Quat Geochronol. 2009; 4: 204-230.

27. Neudorf, CM, Roberts, RG, Jacobs, Z. Sources of overdispersion in a K-rich feldspar sample from north-central India: Insights from De, K content and IRSL age distributions for individual grains. Radiat Meas. 2012; 47: 696-702.
